# Copper oxide nanoparticle toxicity profiling using untargeted metabolomics

**DOI:** 10.1186/s12989-016-0160-6

**Published:** 2016-09-08

**Authors:** Matthew S. P. Boyles, Christina Ranninger, Roland Reischl, Marc Rurik, Richard Tessadri, Oliver Kohlbacher, Albert Duschl, Christian G. Huber

**Affiliations:** 1 Department of Molecular Biology, Division of Allergy and Immunology, University of Salzburg, Hellbrunner Strasse 34, 5020 Salzburg, Austria; 2 Department of Molecular Biology, Division of Chemistry and Bioanalytics, University of Salzburg, Hellbrunner Strasse 34, 5020 Salzburg, Austria; 3 Center for Bioinformatics, University of Tübingen, Tübingen, Germany; 4 Department of Computer Science, University of Tübingen, Sand 14, 72076 Tübingen, Germany; 5 Faculty of Geo- and Atmospheric Science, Institute of Mineralogy and Petrography, University of Innsbruck, Innrain 52, 6020 Innsbruck, Austria; 6 Quantitative Biology Center, University of Tübingen, Auf der Morgenstelle 10, 72076 Tübingen, Germany; 7 Faculty of Medicine, University of Tübingen, Geissweg 3, 72076 Tübingen, Germany; 8 Max Planck Institute for Developmental Biology, Spemannstraße 35, 72076 Tübingen, Germany

**Keywords:** Untargeted metabolomics, Copper oxide nanoparticles, Apoptosis, Oxidative stress, Toxicity profiling, Adverse outcome pathways

## Abstract

**Background:**

The rapidly increasing number of engineered nanoparticles (NPs), and products containing NPs, raises concerns for human exposure and safety. With this increasing, and ever changing, catalogue of NPs it is becoming more difficult to adequately assess the toxic potential of new materials in a timely fashion. It is therefore important to develop methods which can provide high-throughput screening of biological responses. The use of omics technologies, including metabolomics, can play a vital role in this process by providing relatively fast, comprehensive, and cost-effective assessment of cellular responses. These techniques thus provide the opportunity to identify specific toxicity pathways and to generate hypotheses on how to reduce or abolish toxicity.

**Results:**

We have used untargeted metabolome analysis to determine differentially expressed metabolites in human lung epithelial cells (A549) exposed to copper oxide nanoparticles (CuO NPs). Toxicity hypotheses were then generated based on the affected pathways, and critically tested using more conventional biochemical and cellular assays. CuO NPs induced regulation of metabolites involved in oxidative stress, hypertonic stress, and apoptosis. The involvement of oxidative stress was clarified more easily than apoptosis, which involved control experiments to confirm specific metabolites that could be used as standard markers for apoptosis; based on this we tentatively propose methylnicotinamide as a generic metabolic marker for apoptosis.

**Conclusions:**

Our findings are well aligned with the current literature on CuO NP toxicity. We thus believe that untargeted metabolomics profiling is a suitable tool for NP toxicity screening and hypothesis generation.

**Electronic supplementary material:**

The online version of this article (doi:10.1186/s12989-016-0160-6) contains supplementary material, which is available to authorized users.

## Background

With the growing catalogue of engineered nanoparticles (NPs) found in consumer products and in approved or under-development therapeutics [1, 2], there are general safety concerns regarding the implications of perceived exposure that may accompany the widening spectrum of NPs in use. Moreover, with the high projected industrial growth of nanotechnology, particularly of metal oxides, there is a real concern for workplace safety, with the relevant workforce potentially at risk from occupational inhalation exposure [3]. During the expansion of these products there has been a conscientious effort to address safety concerns and to determine the risks involved in human exposure. However, the current understanding is still insufficient to provide suitable recommendations for regulatory decisions [4]. This common international goal may be aided by developing methods for high-throughput screening of NPs and methods which allow the collection of multiple biological endpoints in one assessment; in this way, we may be able to address the exponential growth of NPs within the public domain.

The use of omics methodologies in the assessment of the physiological effects may provide a suitable platform and, therefore, has received much interest in evaluating NP-induced toxicity. Comprehensive omics approaches are ideally suited to investigate the multitude of potential effects that NP exposure exerts on cells, tissues or even whole organisms, since they provide a holistic view on what is going on at the protein or at the metabolite level. Proteomics has received much interest and has been used to show the fate of NPs exposed to physiological surroundings such as plasma [5] and the effects of NPs at a cellular [6] or even at the organism level [7]. Somewhat more directly, metabolomics detects phenotype changes and could therefore deliver valuable information on the toxic effects of NP treatment [8]. Two main technical approaches are suitable to comprehensively detect differences on relative metabolite abundances dependent on NP treatment, in an untargeted fashion: nuclear magnetic resonance-based techniques [9–11] and mass spectrometry (MS)-based approaches. In particular MS-based approaches have seen tremendous technological developments and combine a high sensitivity with excellent resolution power. The increasing amount of data generated by the latest generation of instruments in turn requires more and more sophisticated bioinformatics tools for data evaluation [12–14]. The growing comprehensiveness of these profiling experiments has thus raised hopes that it can help uncover molecular mechanisms associated with NP exposures. However, the scarcity of studies utilising metabolomics in nanotoxicology has been highlighted in a recent review by Lv et al. [15], albeit these applications can be quite diverse, with measurements made of metabolites differentially expressed and excreted in urine, found in serum, identified in organs such as the liver, or assessed intracellularly or extracellularly in in vitro studies. The current literature mainly addresses exposure to TiO_2_ and SiO_2_ NPs, but there are also examples of studies on silver-, zinc-, copper-, carbon nanotube-materials (CNTs), amongst others [15]. Assessment of a cells’ whole metabolome provides an assessment of cellular responses at a molecular level [15], and changes within this upon NP exposure can provide a magnitude of information if assessed correctly; it would be possible to assign specific markers for known toxicity paradigms, and, furthermore, give insights into novel intracellular events. This can allow a platform for both high-throughput screening, but also for toxicity hypothesis generation.

For the purpose of this study copper oxide nanoparticles (CuO NPs) were chosen; as extensive studies on the toxicity mechanisms already exist, CuO NPs provide an excellent model particle for use in this study. Furthermore, with tangible potential for exposure the concern for human health is understandable. Historically, copper mines and smelting facilities have presented a high risk of occupational exposure, reported to induce various respiratory complications [16]. Presently CuO NPs are produced on a large-scale basis [17], and although industrial control of these materials has improved, facilities may vary in their exposure levels dependent upon ventilation systems [18]. The reduced size and increased surface area of CuO NPs compared to bulk material results in the NP form having improved thermoconductivity and fluid viscosity, as well as enhanced Cu ion release; this has led to an assortment of applications, including their use as chemo-sensors, in chemical catalysis, as surfactants and antimicrobials. However, these prolific applications may also allow for greater opportunities for human exposure [19]. Copper, mostly in its ionic Cu^2+^ form, is involved in many biological functions, it is an essential trace element and therefore subject to strict homeostatic control [20]. It is, for instance, incorporated into redox active proteins, mainly in the prosthetic groups of enzymes and thus turned over, recycled, and eventually excreted [21–23].

With perceived increases in exposure through manufacturing and recycling of products and increased use of Cu NPs, the toxicity to humans and any safety concerns need to be assessed. The toxicity of CuO NPs has therefore been studied intensively, also because its toxicity is particularly pronounced compared to other NPs [24]. It has been shown in vitro that CuO NPs induce cytotoxicity in numerous cell types [25], including A549 cells [26], often related to apoptosis [26, 27], and occurring concomitantly with genotoxicity [26, 28] and oxidative stress [29, 30]. It has been proposed that the mode of action for CuNP toxicity lies with the delivery and subsequent intracellular dissolution as Cu^2+^, termed a *Trojan horse mechanism* [31]; identified when biological responses to CuO NPs were found to be greater than those to micrometre sized copper particles or to soluble copper chloride (CuCl_2_) [26, 30]. Cytotoxicity of CuO NPs was shown to be reduced when particles were stabilised, and released fewer ions [31]. Both these key pathways, oxidative stress and apoptosis, have also been demonstrated in response to CuO NPs in vivo [32].

Using the A549 (adenocarcinoma human alveolar basal epithelial) cell line, a well-established and frequently used model for the assessment of NP-induced lung toxicity, we have used untargeted metabolomics as a platform for toxicity profiling of CuO NPs and as a tool for hypothesis generation, focussing on two well-reported pathways of CuO NP-induced toxicity, oxidative stress and apoptosis. These hypotheses were subsequently critically tested by targeted follow-up studies assessing the proposed toxicity pathways by dedicated cell assays. In this proof of principle study, it was expected that the CuO NPs that were tested would induce both oxidative stress and apoptosis, and, therefore, that specific markers within the metabolome would be identified as being linked to these toxicity pathways.

We were able to link the generated metabolome profiles generated in A549 cells to mechanisms of toxicity. Furthermore, we have identified specific indicator metabolites for several pathways, including oxidative stress and apoptosis. We were also able to deduce a more detailed mechanism by which CuO NPs trigger these pathways. These findings suggest that untargeted metabolomics can be applied in early screening of NP toxicity and is advantageous for generating toxicity hypotheses which can be validated more specifically using more traditional methods.

## Methods

### Chemicals and materials

CuO NPs were obtained from Intrinsiq Materials Ltd (Farnborough, UK) and were supplied by the Nanovalid consortium (http://www.nanovalid.eu/). Acetonitrile (ACN) for LC-MS was purchased from VWR (Radnor, PA, USA). High-purity water (H_2_O) was produced using a Milli-Q Integral three purification system from Merck Millipore (Darmstadt, Germany). Standard substances used for identification were obtained from Merck (amino acids). Staurosporine (STS) was purchased from Proteinkinase.de (Kassel, Germany), camptothecin (CPT) from Abcam (Cambridge, UK), and rhTNF-α from Immunotools (Friesoythe, Germany). SYBR Green Supermix was purchased from Bio-Rad (Munich, Germany), RevertAid HMinus M-MulV reverse transcriptase from Fermentas (St. Leon-Roth, Germany), TRIzol reagent from Invitrogen, IL-8 ELISA kits from PeproTech, while Celltiter-Blue® (CTB) Cell Viability Assay was purchased from Promega (Madison, WI, USA), foetal calf serum (FCS) from PAA (Pasching, Austria). All other substances used were obtained from Sigma-Aldrich (St. Louis, MO, USA).

### Cell culture and treatment

The A549, human lung alveolar adenocarcinoma cell line, was purchased from ATCC and maintained in 150 cm^2^ flasks using RPMI 1640 medium supplemented with 10 % foetal calf serum (FCS), 1 % L-glutamine, 100 U/ml Penicillin, and 100 μg/ml Streptomycin, at 37 °C and 5 % CO_2_. To subculture, or seed cells for experiments, cells were trypsinized, centrifuged at 320 x g for 5 min, resuspended in cell culture medium (CCM), and viability determined by trypan blue exclusion. Cells were seeded 1 day prior to experiments at 2 x 10^5^ cells per cm^2^ of culture dish, in either 96-, 24-, or 6-well plates, depending upon experimental protocol. A549 cells were exposed to the following agents: CuO NPs, copper chloride (CuCl_2_), CPT, STS (positive control in apoptosis assays), TNF-α (positive control in pro-inflammatory mediator release), and Triton X-100 (positive control in cytotoxicity assays); exposure time and treatment concentration were dependent on assay.

### Characterization of copper oxide nanoparticles

Characterisation was performed at a CuO NP concentration identical to that used in the metabolomics study (10 μg/ml). The hydrodynamic diameter and an estimation of surface charge of CuO NPs were determined by dynamic light scattering (DLS) and zeta potential measurements, respectively, using a Malvern ZetaSizer Nano ZSP (Malvern Instruments, Malvern, UK). CuO NPs were suspended in dH_2_O or CCM at 10 μg/ml and sonicated prior to measurement. As substantial agglomeration was observed in DLS measurements, primary particle size was also determined by transmission electron light microscopy (TEM). CuO NPs, suspended in dH_2_O, were added to TEM grids and dried prior to imaging using a LEO 912 AB Omega transmission electron microscope (Zeiss, Oberkochen), operated at 120 kV with a LaB6 cathode. Size distributions were calculated using ImageJ software (National Institutes of Health, Bethesda, MD, USA), taking at least 1000 measurements. Dissolution of CuO NPs was assessed, in triplicate, in full CCM. NPs were incubated at 10 μg/ml for 0 h, 1 h, 3 h, 6 h, 12 h, 24 h, 48 h, at 37 °C. After incubation, the samples were centrifuged at 25,000 x g for 30 min and the supernatant was used for Cu^2+^ determination by inductively coupled plasma atomic emission spectroscopy (ICP-AES), using an Activa instrument (Horiba Jobin Yvan, Unterhaching, Germany).

*Cytotoxicity and cell viability* were assessed using lactate dehydrogenase (LDH) quantification in the cell supernatant and the CTB Cell Viability Assay, respectively. Three biological replicates were performed. A549 cells were exposed to CuO NPs at 0–40 μg/ml, or to CuCl_2_ at corresponding copper concentrations of 63–500 μM for 4, 24 and 48 h. The concentrations used for CuCl_2_ exposure relate to the absolute maximum potential release of Cu^2+^ from the CuO NPs; based on the dissolution data, the actual release into the cell culture medium would have been significantly lower. CTB reagent was added to cells in CCM at a ratio of 1:5 and incubated at 37 °C for 60 min; following this, fluorescence intensity was measured at ex/em wavelengths of 560 nm/590 nm on a plate reader (Infinity 200 Pro, Tecan, Groedig, Austria). LDH present in the supernatant was determined as a measure of lost membrane integrity, using a method based on a Sigma diagnostic kit, as previously described [33, 34]. Medium only treated cells were used as negative control exposures. In both assays Triton X-100 (0.1 %) was used as a positive control. The supernatants from these exposures were stored at −80 °C for evaluation of secreted interleukin (IL)-8. These data were used to determine appropriate particle exposure concentrations and time points for subsequent assays.

*Pro-inflammatory cytokine secretion* was assessed through determination of IL-8 by ELISA, following an adaption of the manufacturer’s instructions. Briefly, the capture antibody was added to wells of a 96 well plate and left at 4 °C overnight, followed by blocking buffer (1 % BSA in PBS) for 1 h, and subsequently the addition of standards, at 0–2000 pg/ml, and samples for 2 h. Detection antibody, in assay diluent (0.05 % Tween-20 and 0.1 % BSA in PBS), was then added for 2 h, followed by an avidin-HRP conjugate for 30 min. Each of the preceding steps were separated by wash steps. A TMB substrate was then added and the reaction was stopped using 2 M H_2_SO_4_. Colour development was measured on a plate reader (Infinity 200 Pro, Tecan, Groedig, Austria) at 450 nm with a reference wavelength of 650 nm, and IL-8 concentrations determined using standard curves. Medium only treated cells were used as negative control exposures. TNF-α (20 pg/ml) was used as a positive control for IL-8 secretion by A549 cells. Three biological replicates were performed.

### Gene expression of HO-1 and GPX1 induced by CuO NPs

A549 cells were exposed to CuO NPs at 0–40 μg/ml, for time periods relating to the metabolomics study of 1, 3, 6, 12 and 24 h. RNA isolation was performed using TRIzol reagent and cDNA generation with RevertAid H Minus M-MulV reverse transcriptase; each following the manufacturer’s instructions. Quantitative real-time RTPCR (qRT-PCR) was performed using a Rotorgene 3000 (Corbett Research, Mortlake, Australia), with iQ SYBR Green Supermix and the following primers: human GPX1 (Qiagen #PPH00154E); human HO-1 sense, 5’-GCCAAGACTGCGTTCCTGCTCAA-3’, and antisense, 5’-TCGCCACCAGAAAGCTGAGTGTA-3’; and human RPLP0 sense, 5’-GGCACCATTGAAATCCTGAGTGATGTG-3’, and antisense, 5’-TTGCGGACACCCTCCAGGAAG-3’. The large ribosomal protein P0 (RPLP0) was used as a reference gene. Primer specificity was confirmed through assessment of the product melting curves. Quantification of relative mRNA expression levels were calculated in relation to the RPLP0 housekeeping gene using the delta delta method of Pfaffl [35], and normalized to medium only treated cells (negative control). Three biological replicates were performed.

*Glutathione redox state* was determined using the GSH/GSSG-Glo™ Assay (Promega), following manufacturers’ instructions. Briefly, after exposure of A549 cells to 0–40 μg/ml CuO NPs for time periods relating to the metabolomics study of 1, 3, 6, 12 and 24 h, cells were lysed in a passive lysis buffer which contains GSH probe Luciferin-NT, converts all glutathione into reduced GSH, and is therefore used to measure total glutathione; for determination of GSSG, duplicate wells were prepared in which the lysis buffer also contained N-ethylmaleimide (NEM), which binds GSH preventing its detection and therefore only assesses the oxidised GSSG. After lysis, a luciferin generation reagent, containing Glutathione Reaction Buffer, dithiothreitol (DTT) and glutathione S-transferase, was added, followed by the addition of luciferin detection reagent. Luminescence was then measured in white 96-well plates on a plate reader (Infinity 200 Pro, Tecan, Groedig, Austria). Luminescence values were converted to μM GSH concentrations using GSH standard curves (in the case of GSSG this incorporated a consideration that one mole of oxidized GSSG is reduced to two moles of GSH for detection within the assay). Medium only treated cells were used as negative control exposures. Once values for GSH and GSSG had been determined, data were finally expressed as a ratio of GSH/GSSG. Four biological replicates were performed.

*Caspase activity* of A549 cells was determined using the Apo-ONE® Homogeneous Caspase-3/7 Assay (Promega), following the manufacturer’s instructions. Briefly, A549 cells were exposed to CuO NPs (0–20 μg/ml), STS (0–2 μM), or CPT (0–32 μg/ml), for time periods relating to the metabolomics study of 3, 6, 12 and 24 h. After exposure period Caspase-3/7 Reagent was added to each well at a 1:1 volume ratio, and incubated for 2 h prior to measurement on a plate reader (Infinity 200 Pro, Tecan, Groedig, Austria), with fluorescence intensity determined at ex485/em530. Medium only treated cells were used as negative control exposures, and data was expressed as a percent of medium only treated cells. Three biological replicates were performed.

### Untargeted metabolomics profiling of CuO NPs, staurosporine and camptothecin

A549 cells were exposed to 10 μg/ml CuO NPs for 0, 1, 3, 6, 12, and 24 h, 1 μM STS for 0, 3, 6, 12, and 24 h, and 16 μg/ml CPT for 0, 6, 9, 12, and 24 h. Medium only treated cells were used as negative control exposures. The experimental and computational pipeline used for metabolomics profiling is described in [14]; the main steps and the modifications from the original protocol are as follows. Three biological replicates were performed.

For metabolite extraction cell culture medium was removed, the cells were washed twice with ice cold PBS (Sigma-Aldrich), followed by an additional very rapid washing step with ammonium bicarbonate (185 mmol/l, 289 mOsm, pH 7.8; Sigma-Aldrich). To quench metabolism, 750 μL ice cold methanol containing ethylparaben (10 μmol/l) as an internal standard, was added. A sonication and centrifugation step followed, which resulted in a supernatant containing the intracellular metabolites. These samples were diluted 1:5 with MilliQ water (containing 10 μmol/l nitrotyrosine) and 1:2 with acetonitrile (containing 10 μmol/l nitrotyrosine) for reversed-phase (RP-)HPLC-MS and for hydrophilic interaction liquid chromatography (HILIC)-MS analysis, respectively.

### High-performance liquid chromatography-mass spectrometry (HPLC-MS)

In order to increase the yield of identifiable hydrophilic metabolites HILIC separation was carried out in addition to the RP separation which was described in detail in Ranninger et al. [14]. The HILIC-MS measurements were performed with a 150 mm x 2 mm Nucleodur column with 1.8 μm particles (Macherey-Nagel, Düren, Germany). The mobile phase A contained 90 % acetonitrile and 10 % buffer (100 mM ammonium formate pH 3.2 adjusted with formic acid) and mobile phase B consisted of 50 % acetonitrile 40 % MilliQ water and 10 % buffer. Using a flow rate of 300 μl/min, chromatography was performed by starting at 100 % A, holding for 3 min followed by a decrease of A to 0 % in 17 min and holding 0 % for 2 min, finally a re-equilibration step for 8 min at 100 % A was carried out. The column temperature was maintained at 30 °C and 2.7 μl of sample was injected.

The detection was carried out using a quadrupole-Orbitrap mass spectrometer (Model Q Exactive Plus Thermo Fisher Scientific, Bremen, Germany) in the mass range 70–950 *m/z* at a resolution of 70,000. The ESI heater temperature was set to 350 °C for RP separation and to 250–300 °C for HILIC; an AGC-target of 1 × 10^6^ and a minimum injection time of 50 ms were chosen. The settings of the tuning parameters for each separation and ionization mode are summarized in Additional file 1: Table S1. The data was recorded in profile mode.

The tandem spectra were acquired in data-dependent mode, by using a top 5 experiment. Therefore the resolution was set to 17,500, the AGC-target to 5 × 10^4^ and a maximum injection time of 64 ms was selected. For fragmentation the normalized collision energy (NCE) was ramped in three steps (20, 40, 60). Furthermore an isolation window of 0.8 *m/z*, an underfill ratio of 1.5 %, an intensity threshold of 1.2 × 10^4^, and an apex trigger of 2 to 5 s were selected. Additionally, *m/z* values with a charge higher than 3 as well as isotopes were excluded for fragmentation. The fragmentation data was recorded in centroid mode.

### Data evaluation for cytotoxicity, cell viability, pro-inflammatory responses, glutathione redox state, gene expression, and apoptosis assays

Statistical analysis of data was performed using PASW statistics 18 (SPSS, IBM), and treatments were considered statistically significant when *p* < 0.05. Differences between exposures of medium only and different concentrations of CuO NPs, CuCl_2_, STS or CPT were determined by ANOVA, with post hoc Tukey comparisons for pairwise analysis. Specific comparisons of CuO NPs versus CuCl_2_ were made using an independent samples *t*-test.

### Data evaluation for untargeted metabolomics

Both chromatograms and mass spectra were recorded with Xcalibur 3.0.63 (Thermo Fisher Scientific). The generated HPLC-MS raw data was converted to mzML data with ProteoWizard 3.0.4243 (http://proteowizard.sourceforge.net/, [36]). All mzML files belonging to one separation and ionization mode were further processed together using OpenMS 1.11 (www.OpenMS.de, [37]), KNIME 2.11.2 (https://www.knime.org/, [38]), R 2.15.1 (www.r-project.org, [39]), and SIMCA 13.0.3 (Umetrics, Umea, Sweden).

Metabolomics data was normalized to total cell number as follows. For each time point and condition the mean cell number was calculated. As trypsinization is known to introduce artefacts in HPLC-MS assays, an additional set of cells, with identical treatments were prepared. Cell counting was performed using trypan blue exclusion on a haemocytometer. The resulting cell numbers are shown in Additional file 1: Tables S2 and S3.

An important aspect in metabolomics data interpretation in regards to in vitro toxicity testing relates to how to prepare data for pattern recognition and biological interpretation. For this it is necessary to monitor cell viability to ensure that sub-lethal particle concentrations are used, or, if necessary, to account for differences in cell numbers, as the exposure of cells to a toxic substance can lead to cell growth arrest or cell death, while control (untreated) cells would be viable and therefore growing and dividing. This can result in different viable cell numbers between control and treated groups, distorting the relative comparison between them. This may influence data interpretation through a drop in a specific metabolite being perceived as something other than a simple drop in cell number, or, conversely, a rise in a specific metabolite not being detected as the overall viable cell number was lower. Therefore, the normalization of detected metabolites is essential in order to correctly determine whether the observed differences in the metabolite profiles is due to the treatment and not to varying cell numbers. Various normalization methods can be applied in metabolomics, such as the wet weight of cells, the cell number, protein or DNA concentration, or even using a comparison to “housekeeping” metabolites [40].

For this CuO NP study all these parameters for normalization were evaluated (data not shown) using cells seeded at different densities to assess how the above mentioned parameters are changed dependent of cell number; which were then further compared to untreated and CuO NP-treated cells after a 24 h exposure period. Although it is a frequently used method for normalization, TIC normalization did not correspond well to cell number. Instead, we found the use of a housekeeping metabolite, namely N-acetylaspartylglutamic acid, particularly effective. However, this is a laborious procedure, as a housekeeping metabolite has to be evaluated for each cell system as well as for the treatment and the study design separately. We consider normalization to total DNA an elegant solution, although, again, this is a long and laborious method. Furthermore, in our hands, the patterns of detection were not consistent within the control experiments performed, which may be due to an inadequate DNA extraction and measurement protocol. Ultimately, the normalization to total cell number was chosen. The metabolic expression patterns were found to be consistent with our control experiments, and as the experimental time and complexity of this method is relatively low, it was considered the most appropriate option to provide a time- and cost-effective method for unbiased determination of the cells’ metabolome.

The data evaluation was carried out according to the procedure described in [14] with minor changes mentioned below. The OpenMS tool FeatureFinderMetabo has the option to set a noise threshold, which varies a lot depending on the separation method, the MS instrument, the ionization mode as well as on the sample. The following settings for the noise threshold were used for the various metabolomics datasets CuO_RP_pos_intracellular 100,000; CuO_RP_neg_intracellular 20,000; CuO_HILIC_pos_intracellular 150,000; CuO_HILIC_neg_intracellular 50,000; Apoptosis_RP_pos: 30,000; Apoptosis_HILIC_neg: 30,000.

From the resulting OpenMS feature list we removed chemical noise and background signals: for each detected feature the median intensity in the blank was not allowed to exceed 20 % of the sample intensity for 30 out of 36 samples. Subsequently the relative standard deviation (RSD) of the two internal standards ethylparaben and nitrotyrosine was evaluated, which had to be less than 20 % in all samples in order to be used for differential analysis (Additional file 1: Table S4). These features were normalized using the cell number, which was acquired in an additional experiment (see Additional file 1: Tables S2 and S3). The peak area of each feature was divided by the according mean cell number in order to account for cell growth and death occurring during the 24 h of treatment. Furthermore, only *m/z* values were used for statistical analysis, which were stable in the pool sample as demonstrated by less than 25 % RSD in peak areas.

The feature numbers gained in the different steps of raw data processing are collected in Additional file 1: Table S5. The features detected by HILIC- and RP-HPLC-HRMS after QC filtering were compared using the exact mass information that had to match within ±5 ppm. The result is illustrated in a Venn diagram (Additional file 1: Figure S1) and shows just a small overlap between the two separation modes. Using both separation methods a greater number of features could be detected allowing a more comprehensive analysis of the metabolic signature.

Data was statistically evaluated using principal component analysis (PCA), and a univariate statistical approach, namely linear models for microarray data (limma) followed by Benjamini-Hochberg correction for multiple testing. The differentially expressed features were identified, whereas the reliability of the assigned identification is indicated as proposed by the guidelines of Sumner et al. [41].

Significantly regulated features were identified by searching the exact feature masses with a maximum deviation of 5 ppm in the human metabolite database (HMDB). Subsequently, confirmation of these putative hits was done by MS^2^ fragment spectra comparison with databases such as Metlin [42] (level 2). Finally, if available, the measurement of reference compounds enabled the comparison of chromatographic retention times, exact mass (level 1a) and the fragmentation patterns (level 1) of the reference compound and the metabolite to be identified.

## Results

### Characterization of CuO nanoparticles

**Fig. 1:**
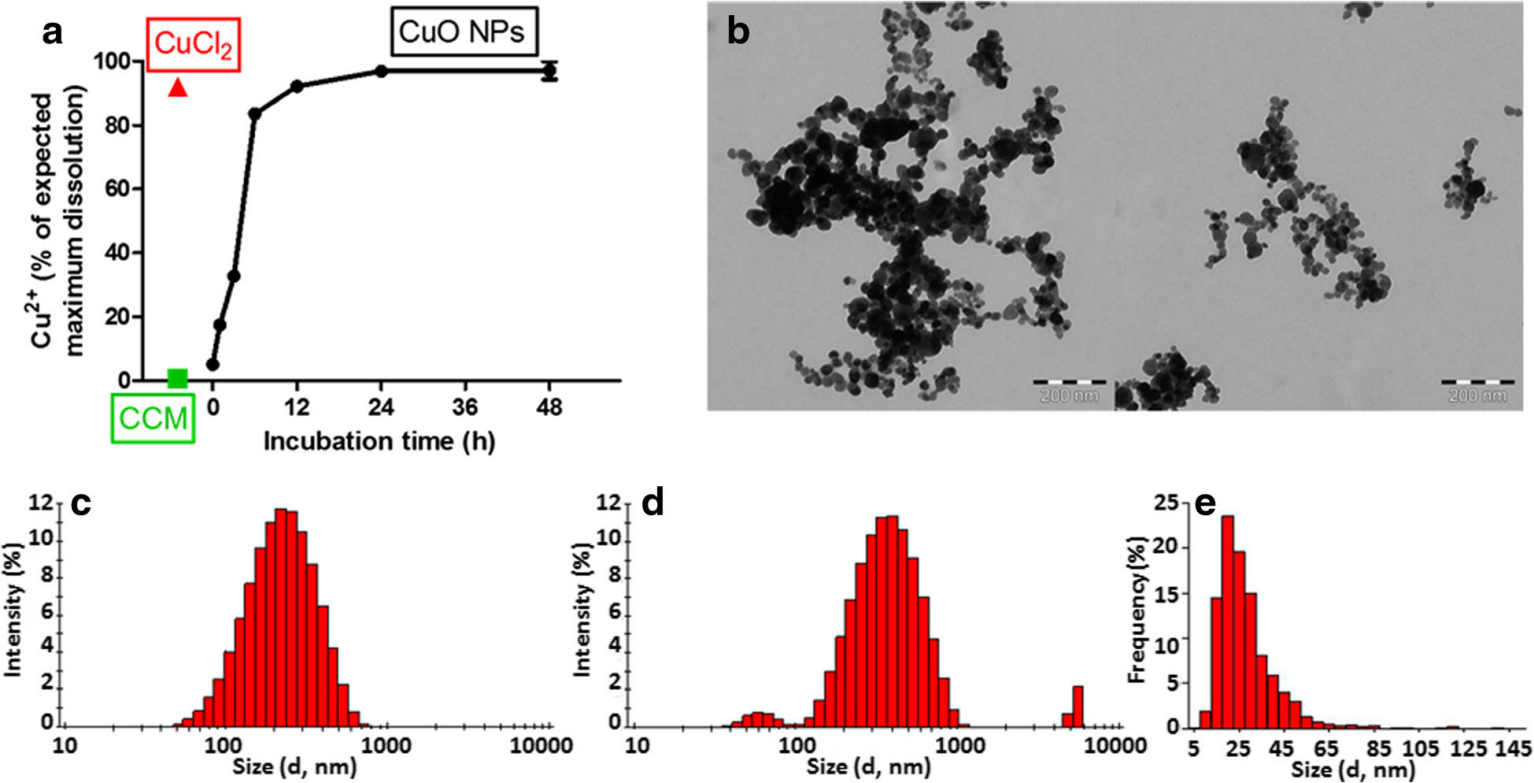
Characterization of CuO NPs. Including (**a**) dissolution of 10 μg/ml CuO NPs (maximum potential Cu ion release = 8 μg/ml, and would be represented as 100 % dissolution), *black* entries represent Cu ions detected after incubation of CuO NPs in CCM for increasing lengths of time, *green* represents Cu ions detected in CCM alone, *red* represents CuCl_2_ control; **b** transmission electron microscopy (TEM) micrographs of CuO NPs, scale bars are shown at bottom right of each image; **c** size distribution histogram of CuO NP hydrodynamic diameter when suspended in water, determined by DLS; **d** size distribution histogram of CuO NP hydrodynamic diameter when suspended in CCM, determined by DLS; **e** size distribution histogram of CuO NP diameter, determined by TEM

CuO NP characteristics are given in Table 1 and Fig. 1. The primary particle size was determined to be 28.2 nm (±13.7) by TEM. However, agglomerates of 172 nm (±7.6) or 214.2 nm (±14.8) were shown to form when CuO NPs were suspended in water or CCM, respectively. In CCM the particle suspension was shown to contain three distinct peaks of agglomerate sizes (Fig. 1d), also highlighted by a high polydispersity index (PDI) of 0.507; in water the PDI was far lower, at 0.188, and only one size distribution peak was observed (Fig. 1c). When in water, the CuO NPs presented a positive surface charge of +35.9 mV (±1.3), which became negative when suspended in CCM (−10.5 mV ±0.1); which is likely due to the coating of NPs with protein components of the CCM, as has previously been shown [34]. As CuO NPs are metal oxide NPs which possess the capacity to release ions, the concentration of Cu^2+^ ions in solution was also assessed (Fig. 1a). The dissolution was studied under the same conditions as those of the cellular experiments, therefore within our CCM and during a time course of 0, 1, 3, 6, 12, 24 and 48 h. We observed a time dependent dissolution. A rapid dissolution was observed between 3 and 6 h. To correlate with the metabolome experiments, a CuO NP concentration of 10 μg/ml was used, therefore a concentration of Cu^2+^ ions equivalent to 8 μg/ml was expected if the NPs dissolved completely; which was observed within 24 h of incubation. CCM served as negative control, and was found to be < 0.05 μg/ml, and as positive control a copper (II) chloride (CuCl_2_ · 2H_2_O) solution was used, this was suspended at 21.45 μg/ml to give a final copper concentration of 8 μg/ml. Although optimal CuO dissolution has been shown under highly acidic or highly basic conditions [43], we have shown, as have others [28], that CuO NPs release significant levels of Cu^2+^ ions in buffered CCM.

**Table 1:** Copper oxide nanoparticle properties including source of material, surface charge in water and in cell culture medium (CCM), and size determined by DLS in water and in CCM, and by transmission electron microscope micrographs

Properties	CuO NPs
Source	Intrinsiq
Zeta potential in water (mV)	+35.9 ± 1.3
Zeta potential in CCM (mV)	−10.5 ± 0.1
Size (determined by DLS) in water (d.nm) (Z-av.)	172.0 ± 7.6 (PDI 0.188)
Size (determined by DLS) in CCM (d.nm) (Z-av.)	214.2 ± 14.8 (PDI 0.507)
TEM size (nm)	28.2 ± 13.7

### Determination of appropriate dose for untargeted metabolomics study, using cytotoxicity and IL-8 secretion induced in A549 cells

**Fig. 2:**
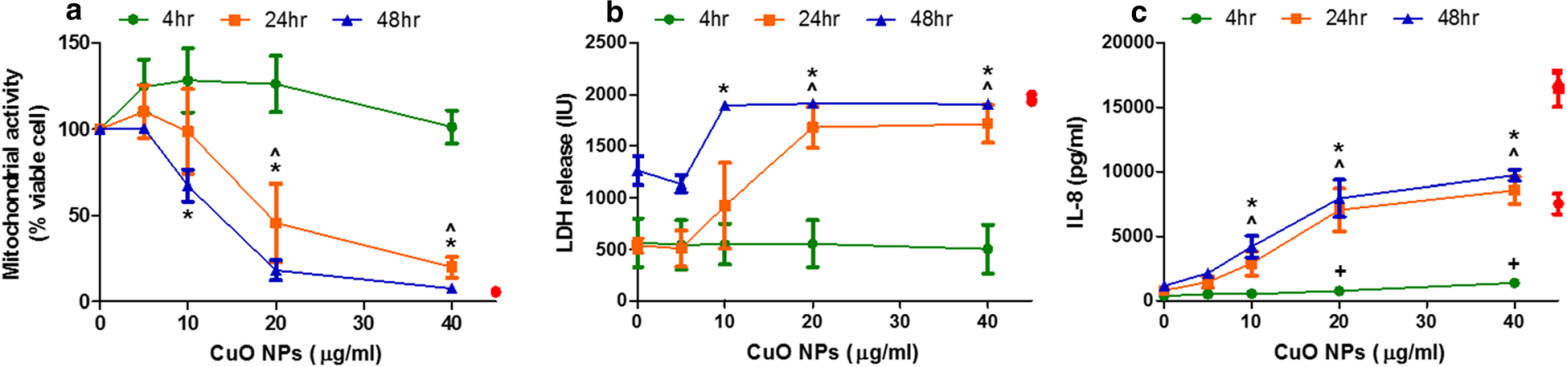
Cell viability, cytotoxicity and IL-8 secretion in response to CuO NPs. Dose finding experiments for the untargeted metabolomics profiling. A549 cells treated with CuO NPs at 0, 2.5, 5.0, 10, 20 and 40 μg/ml for 4, 24 and 48 h and assessed for (**a**) cell viability (**b**) LDH release, **c** IL-8 secretion. Each data point represents the mean ± SEM (*n* = 3). Statistical significance was determined by ANOVA with Tukey posthoc, and identified with + for 4 h exposures, ^ for 24 h, and * for 48 h, when *p* < 0.05 compared to medium only treated cells. Positive controls used were Triton X-100 for viability and cytotoxicity assays, and TNF-α for IL-8 secretion (appear in *red* on each graph)

The concentration selection as well as the exposure period for the untargeted metabolomics profiling of A549 cells was determined by testing cell viability, cytotoxicity, and IL-8 secretion in response to a CuO NP concentration range of 0–40 μg/ml for 4, 24 and 48 h, the results are shown in Fig. 2. Almost complete cell death was induced with exposures of ≥ 20 μg/ml for 24 h and above, with a strong and statistically significant IL-8 secretion observed from exposures of ≥ 10 μg/ml during the 24 and 48 h exposure periods. A final concentration of 10 μg/ml, with 24 h exposure, was selected for subsequent metabolomics assays. Assessment of A549 cells in response to CuO NPs in comparison to CuCl_2_ at corresponding copper concentrations of 63–500 μM, was performed for the same biological endpoints described above. A similar pattern of dose- and time-dependent cytotoxicity and IL-8 secretion was observed for each material. However, these responses were shown to be greater in response to CuO NPs than to corresponding exposures of CuCl_2_ (Additional file 1: Figure S2).

### Differential metabolome analysis of A549 cells in response to CuO NPs

**Fig. 3:**
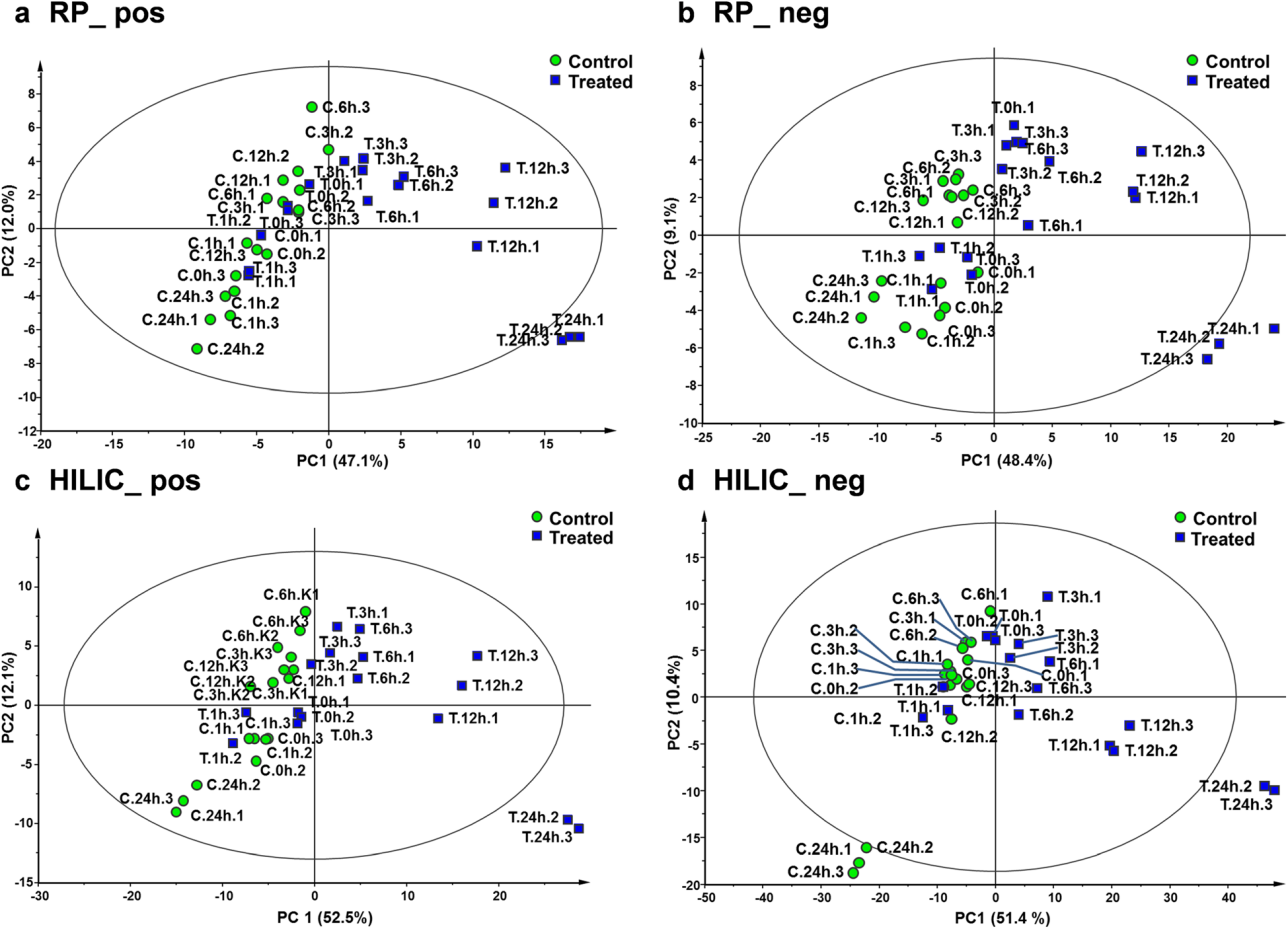
Principal Component Analysis Score plots showing controls (C) and treated samples (T) for the different time points (0 h, 1 h, 3 h, 6 h, 12 h, 24 h) and the biological replicates independently (*n* = 3) as datasets 1, 2, and 3. **a** reversed-phase HPLC - & positive ionization (RP_pos): R^2^cum (0.591),Q^2^cum (0.481); **b** reversed-phase HPLC & negative ionization (RP_neg): R^2^cum (0.575),Q^2^cum (0.459); **c** HILIC & positive ionization (pos) (without T.1 h.1, T.24 h.1): R^2^cum (0.732),Q^2^cum (0.586); **d** HILIC & negative ionization (neg) (without T.24 h.1): R^2^cum (0.618),Q^2^cum (0.542)

In order to profile the time-dependent responses of metabolites upon exposure of A549 cells with 10 μg/ml CuO NPs, six time points (0, 1, 3, 6, 12 and 24 h) were tested and the cells’ metabolome was assessed in CuO NP treated cells and cells incubated for the same time period in CCM only. Principle component analysis (PCA) is a statistical tool that can be used to visually explore the grouping pattern of all samples of the study, where R^2^ and Q^2^ are used for evaluating the performance of the PCA model. The PCA score plots for the CuO NP study demonstrated that in all four methods used, chromatographic separation with reversed-phase high-performance liquid chromatography (RP-HPLC)/hydrophilic interaction liquid chromatography (HILIC) and negative (neg)/positive (pos) electrospray ionization followed by mass spectrometric detection, CuO NP exposure induced a separation in the grouping of regulated metabolites from the untreated cells after 6, 12 and 24 h (Fig. 3). Taking into consideration the comprehensiveness of the features detected by all methods together (Additional file 1: Figure S1), the holistic view of metabolic processes influenced by CuO NP treatment is well supported.

**Fig. 4:**
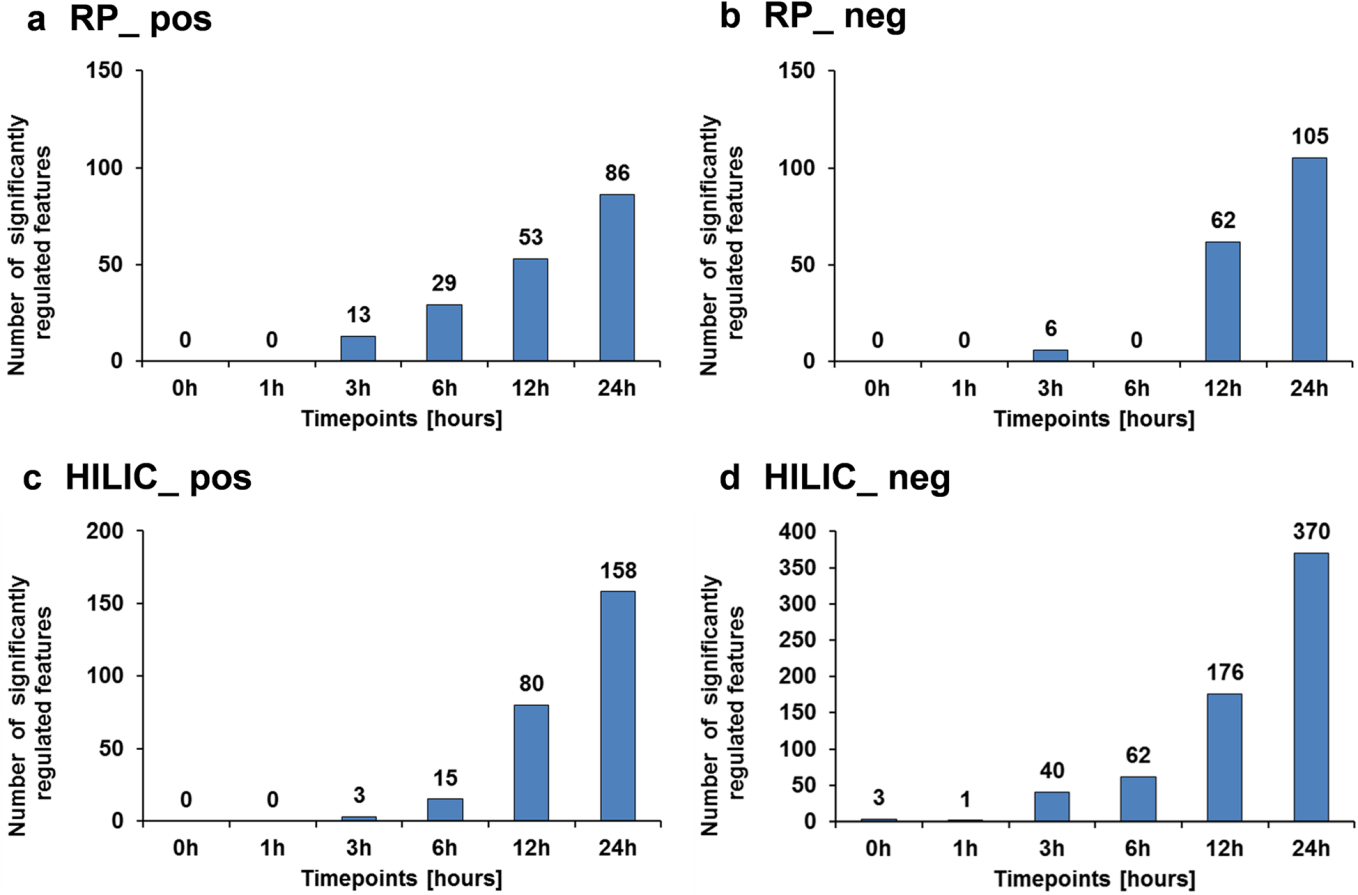
Number of significantly regulated features (*p* < 0.05) calculated using limma followed by Benjamini-Hochberg correction for multiple testing. (**a**) reversed-phase HPLC & positive ionization (RP_pos) (**b**) reversed-phase HPLC & negative ionization (RP_neg) (**c**) HILIC & positive ionization (HILIC_pos) (**d**) HILIC & negative ionization (HILIC_neg)

A similar result was observed when using the univariate statistical approach. The pairwise comparison of control versus treated for each time point confirmed that significantly regulated features were observed only for the later time points (Fig. 4). Moreover, it can be seen that through the time course of the experiment the numbers in differentially regulated features continually increased, highlighting the pronounced changes induced by CuO NPs at a metabolite level. In order to perform a biological interpretation, it is necessary to turn the (differentially expressed) features (m/z values) into identified metabolites. This identification process is one of the major bottlenecks in metabolomics studies. The reliability of the assigned identification depends on how much analytical information of a feature is available. Therefore different levels of identification (levels 1, 1a and 2) have been proposed by Sumner et al. [41]. Accordingly, 34 differentially regulated metabolites were identified in total according to level 1, 1a and 2 [41], which were subsequently used for biological interpretation (Additional file 1: Table S6).

The majority of the significantly regulated features we found were not unambiguously identified and were therefore not included in Additional file 1: Table S6, which contained 34 differentially expressed metabolites. In our case, an unambiguous identification needed a reference substance or a database reference tandem mass spectrum that was highly similar to our acquired tandem mass spectrum. The total number of features that were differentially expressed upon CuO NP treatment is shown in Additional file 1: Table S7. Amongst these features were several candidates that were too low in abundance to trigger a tandem mass spectrum and therefore no unambiguous identification was possible. Furthermore, some features could not be identified, as the information gained in an HPLC-MS approach was insufficient for identification, for example for phosphatidylethanolamines (PE), phosphatidylserine (PS), and phosphatidylcholines (PC). These substance classes do seem to be affected upon CuO NP treatment, however, they were detected mainly using HILIC-negative ionization, which, in general, was a separation method resulting in a relatively high number of differentially expressed features.

The number of differentially expressed features (Additional file 1: Table S7) is quite high when compared to the number of unambiguously identified metabolites (Additional file 1: Table S6), however, the reliability of those reported in Additional file 1: Table S6 appears to be high, as indicated within the table.

The differentially regulated metabolites shown in Additional file 1: Table S6 belong to different classes and play roles in various pathways. One possible approach for biological interpretation is the use of pathway mapping software. In this case Ingenuity Pathway Analysis (IPA) [44] was used. The most affected cellular functions were amino acid metabolism followed by molecular transport, small molecule biochemistry, cellular growth & proliferation, and organismal development. However, these interpretations can only be generalised to the overall output of the cells. For the purposes of this study it was important to determine specific pathways which relate to the toxicity of CuO NPs, and therefore a focus was given to oxidative stress and apoptosis. Metabolites identified, such as glutathione, cysteine-glutathione disulfide, and citrulline were considered to be associated with oxidative stress; while others, including glycerophosphocholine (GPC), numerous amino acids, myo-inositol, and 5’-methylthioadenosine (MTA) were considered to be associated with apoptosis. These points led to our “hypothesis” that CuO NPs induce both oxidative stress and apoptosis. These points are addressed within the discussion, and warranted further testing using conventional assays to confirm both oxidative stress and apoptosis.

### Validation of metabolomics data using assessment of oxidative stress

**Fig. 5:**
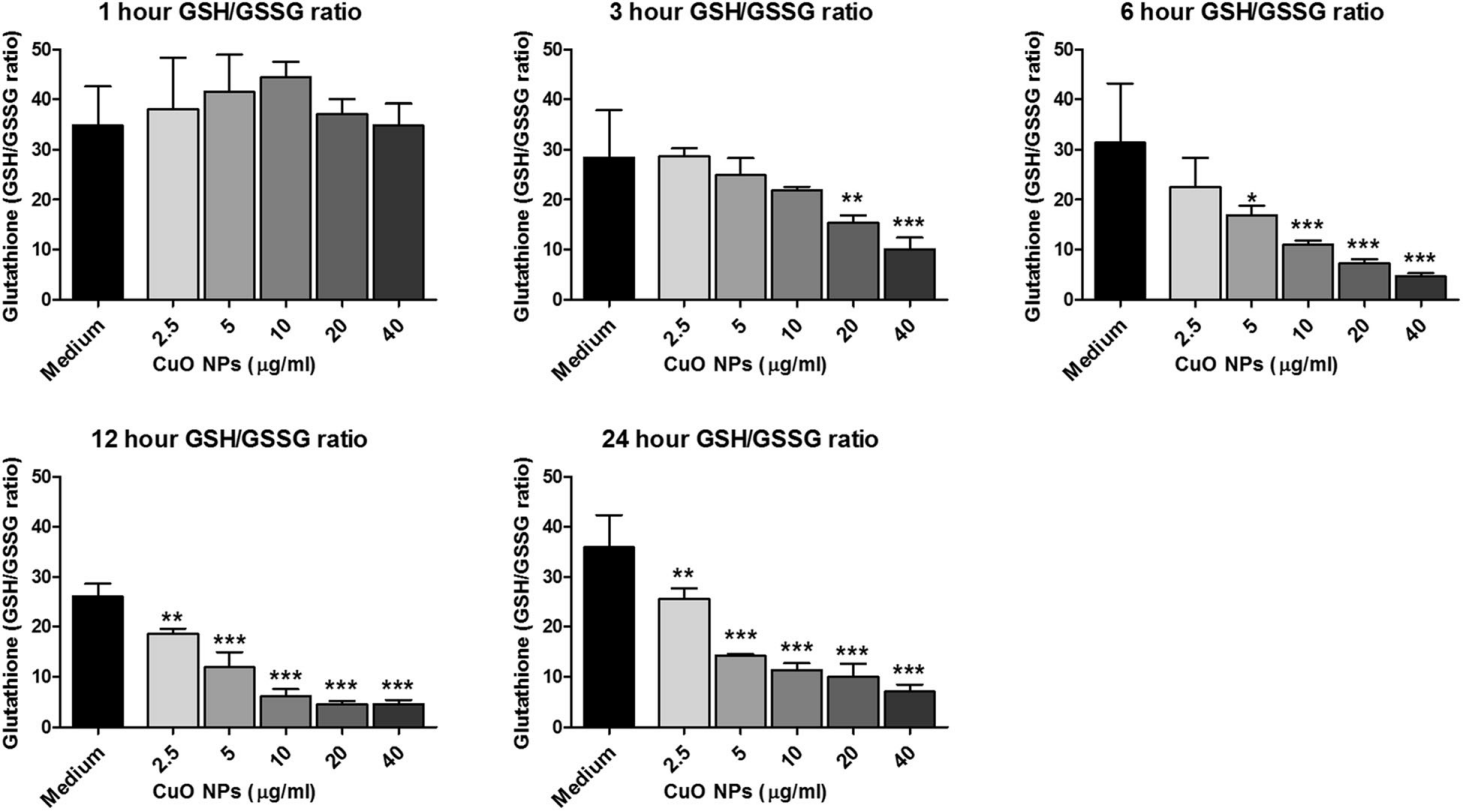
Glutathione redox state in response to CuO NPs. A549 cells treated with CuO NPs at 0, 2.5, 5.0, 10, 20 and 40 μg/ml for 1, 3, 6, 12 and 24 h. Data is expressed as GSH/GSSG ratio, and each data point represents the mean ± SEM (*n* = 4). Statistical significance (determined by ANOVA with Tukey posthoc) is shown by * = *p* < 0.05, ** = *p* < 0.01, *** = *p* < 0.005, when compared to medium only treated cells

To determine if the trend of differently regulated metabolites related to oxidative stress, which appeared only after 6 h incubation, A549 cells were exposed to 0–40 μg/ml CuO NPs for 1, 3, 6, 12 and 24 h. The intracellular GSH/GSSG ratios were assessed using a luminescence-based method to detect total glutathione and oxidised GSSG. This method permitted the fast isolation of GSSG and therefore allowed to determine accurate GSH/GSSG ratios. A dose- and time-dependent decrease in GSH/GSSG ratio was observed (Fig. 5). Significant oxidation of glutathione occurred in as early as 3 h with concentrations of 20 and 40 μg/ml, while all concentrations tested induced significant glutathione oxidation during the latter two time points. When considering the concentration used in the metabolomics study, 10 μg/ml, it was evident that a significant oxidation of glutathione only occurred after 6 h, and remained for the duration of the experiment. Assessment of the cells’ response to a redox imbalance at this time point was further corroborated with hemeoxygenase-1 (HO-1) mRNA expression (Additional file 1: Figure S3), which, although significantly upregulated after 3 h in response to the highest two concentrations tested, was only found in response to 10 μg/ml after 6 h. Glutathione peroxidase 1 (GPX1) mRNA expression was also assessed under the same conditions (Additional file 1: Figure S4). It was evident that only the highest exposure concentrations, and predominantly the longest exposure time of 24 h were sufficient for inducing gene expression of this antioxidant.

### Validation of metabolomics data using assessment of apoptosis

**Fig. 6:**
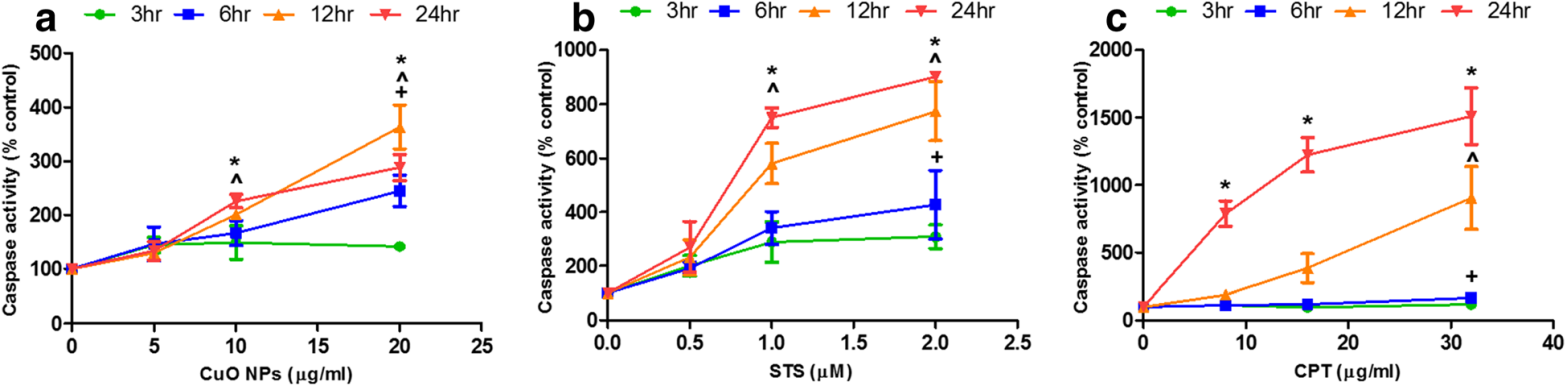
Apoptosis (caspase-3 and −7 activity) in response to CuO NPs, staurosporine (STS) and camptothecin (CPT). A549 cells treated with (**a**) CuO NPs at 0, 2.5, 5.0, 10 and 20 μg/ml, (**b**) STS at 0.5, 1, and 2 μM, or (**c**) CPT at 8, 16, and 32 μg/ml; for 3, 6, 12 and 24 h and assessed for caspase-3 and −7 activity. Data is expressed as % enzyme activity compared to medium only treated cells, and each data point represents the mean ± SEM (*n* = 3). Statistical significance was determined by ANOVA with Tukey posthoc, and + for 6 h, ^ for 12 h and * for 24 h, when *p* < 0.05 compared to medium only treated cells

Caspase-3 and −7 activity was assessed in response to CuO NPs at 0–20 μg/ml (Fig. 6a), and to two further substances, namely staurosporine (STS) (Fig. 6b) and camptothecin (CPT) (Fig. 6c). The addition of the second two substances (STS and CPT) would allow us to gain confidence in metabolic markers which we may propose as generic markers for apoptosis. Cells were exposed to 0.5, 1, and 2 μM STS, or 8, 16, and 32 μg/ml CPT, for 3, 6, 12, and 24 h. A dose- and time-dependent increase in caspase activity was observed with exposure of CuO NPs, with significant increases found in response to 10 μg/ml (12 and 24 h) and 20 μg/ml (6, 12 and 24 h) CuO NPs. STS induced a clear dose- and time-dependent increase in caspase activity when compared to untreated cells, with significantly increased activity in response to 1 μM (12 and 24 h) and 2 μM (6, 12, and 24 h). The response to CPT was similar, although increased activity was observed at all concentrations tested, 8 μg/ml (24 h), 16 μg/ml (24 h), 32 μg/ml (6, 12, and 24 h). However, although both substances induced apoptosis, indicated by a strong caspase activity, STS was shown to induce an early activation of caspases at low concentrations (in respect to the dose range used here), while CPT needed higher concentrations (in respect to the dose range used here) and a clear dose response was only observed during the latter time points. Therefore, these response characteristics were taken into account for the study design of the subsequent metabolomics experiment.

### Differential metabolome analysis of STS and CPT, and comparisons to CuO NPs

Untargeted metabolomics was used to compare the differentially expressed metabolites of untreated cells to those expressed with treatments of STS and CPT, with exposure times of 0, 3, 6, 12, 24 h for STS and 0, 6, 9, 12, 24 h for CPT, and concentrations of 1 μM STS (0.5 μg/ml) and 16 μg/ml CPT.

**Fig. 7:**
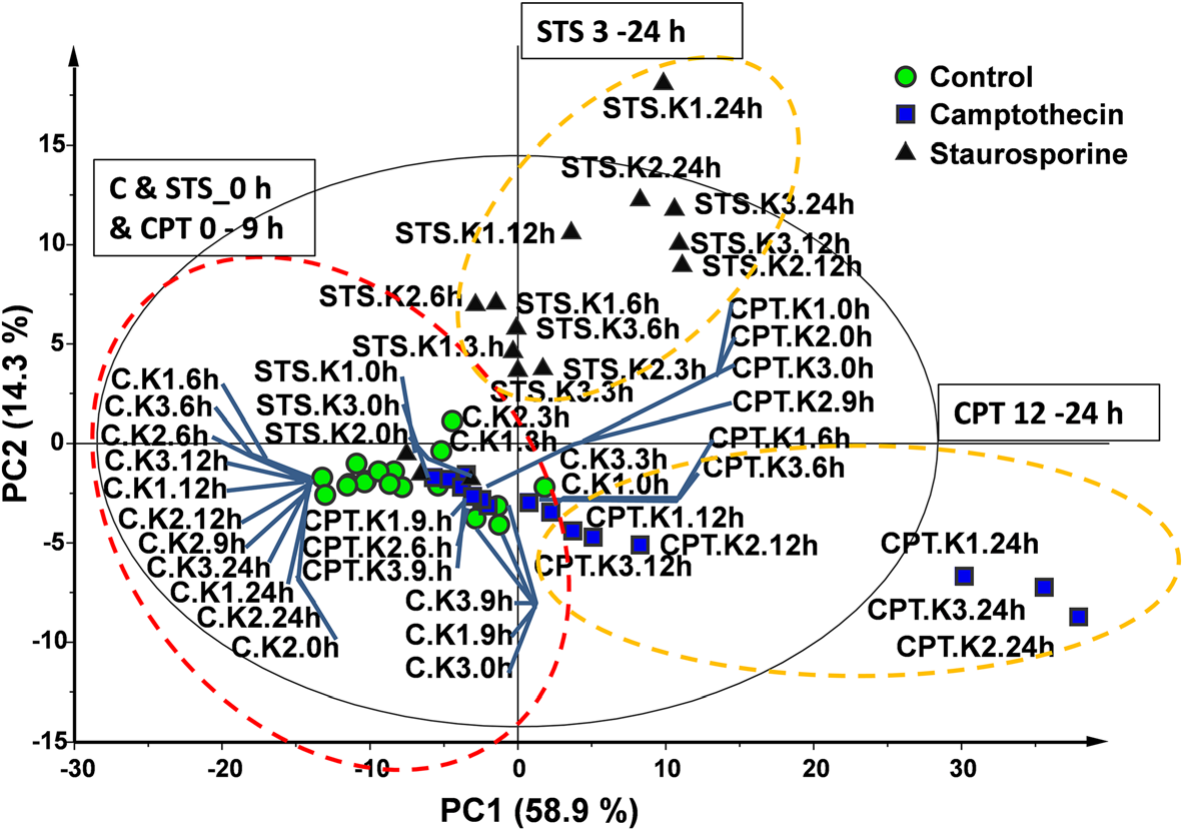
Principal Component Analysis score plot showing controls (C), staurosporine treated samples (STS) and camptothecin treated samples (CPT) for the different time points (0 h, 3 h, 6 h, 9 h 12 h, 24 h) and biological replicates shown independently (*n* = 3, identified with K1,K2, and K3) for the HILIC negative dataset (R^2^(cum) 0.731 and Q^2^ (cum) 0.684)

The metabolomics data generation and processing were carried out in accordance to the CuO NP toxicity study, again including data normalized to cell number (Additional file 1: Table S3). The statistical evaluation of the metabolomics data detected similar trends to those observed in the caspase activity assays. Limma as well as PCA identified that differences between control and treated samples occurred from 6 h onwards for STS, while for CPT differences were observed from 12 h onwards. Although the treatment with STS induced metabolic changes more rapidly, after 24 h of exposure more metabolic features were differentially expressed upon incubation with CPT than STS, this is visualized by the grouping in the principal component analysis plots (Fig. 7). Notably, this approach signalled that, although both STS and CPT induced apoptosis in A549 cells, different metabolites were involved.

**Fig. 8:**
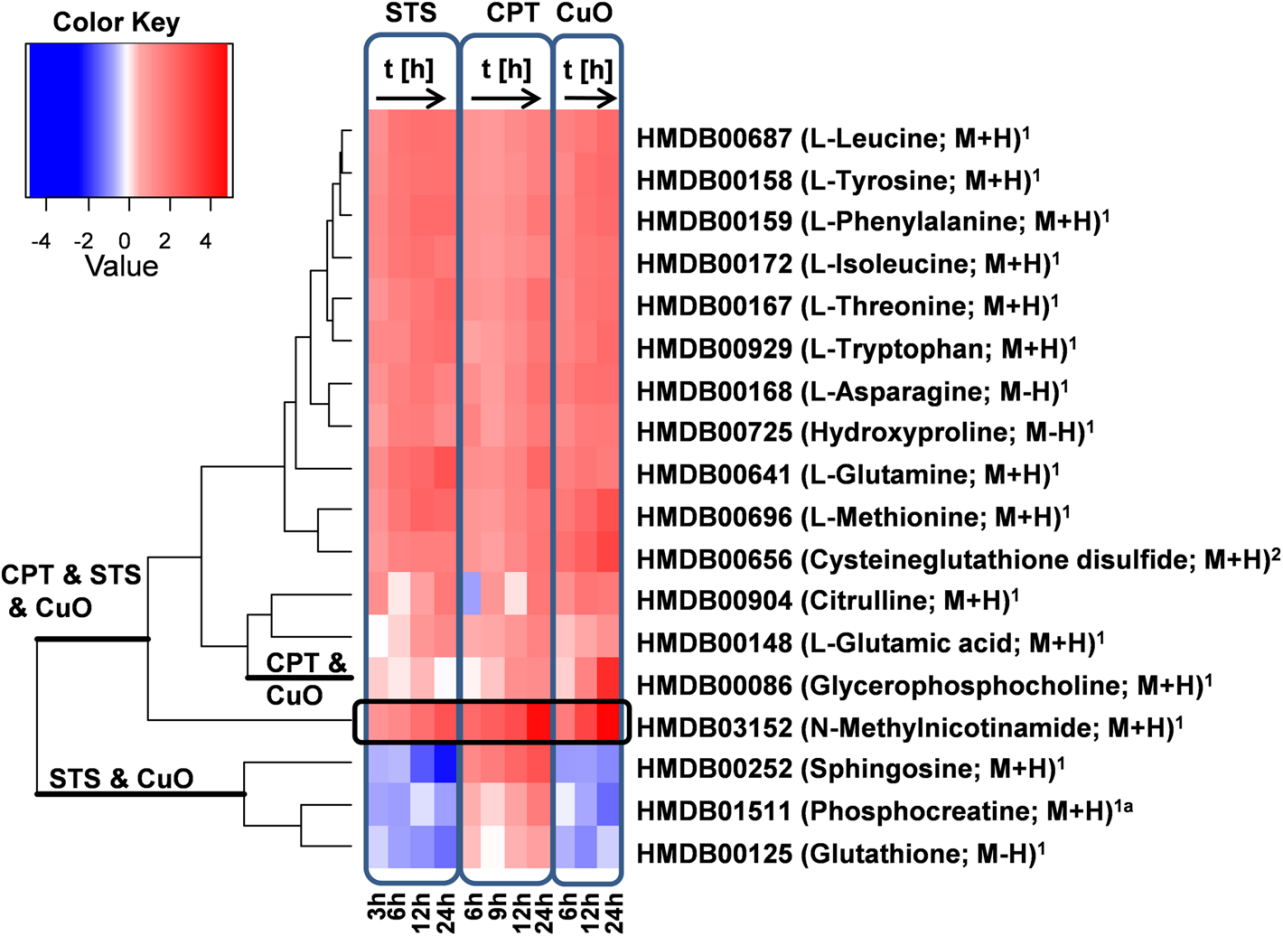
Heatmap showing log_2_ fold changes for treatments of staurosporine (STS), camptothecin (CPT) and copper oxide nanoparticles (CuO NPs) for the various time points (*n* = 3), the levels of identification are indicated by ^1^identified by reference substance (MS^2^ and RT), ^1a^identified by reference substance (only RT), ^2^identified by comparison of MS^2^ spectra with reference databases such as Metlin. The dendrogram on the left hand side shows a clustering of similarly regulated metabolites upon treatment with STS/CPT/CuO NPs

All three apoptotic agents, CuO NPs, STS, and CPT, induced a differential expression of metabolites, presented as a heat map in Fig. 8. However, although frequently similar, the dendrograms indicated that the induction patterns generated by each agent were also often markedly different. The colour coding in Fig. 8 indicates the extent of upregulation (red) or downregulation (blue) of a certain metabolite and further elucidates trends, such as successive downregulation of sphingosine over time upon treatment with STS. An accumulation of the various amino acids was observed for all three treatments. However, although a strong time-dependent upregulation was observed in response to CuO NPs, MTA was unaltered in response to STS or CPT. Furthermore, GPC regulation in response to STS was shown to have no association with exposure time, while for both CuO NPs and CPT a strong time-dependency was evident. Other metabolites differentially regulated in all three treatments included glutathione, sphingosine, phosphocreatine, and methylnicotinamide (MNA).

In most cases, however, uniformity of response was not observed, as glutathione was increased in response to CPT and decreased in response to CuO NPs and STS. A strong time-dependent decrease in sphingosine was observed in response to STS, while CPT induced a strong time-dependent increase and CuO NPs, although decreasing sphingosine, did so independent of exposure time. Additionally, phosphocreatine was shown to increase in response to CPT, and decrease in response to STS and CuO NPs. However, MNA was found to be strongly and time-dependently up-regulated within all three treatments (Additional file 1: Figure S5).

## Discussion

### Toxicity of CuO NPs in A549 cells

In experiments used to determine the appropriate dose for untargeted metabolomics study, including cytotoxicity and IL-8 secretion, CuO NPs were found to induce almost complete cell death in A549 cells when exposed with concentrations of 20 μg/ml or greater, by the 24 h exposure interval. At the lower concentration of 10 μg/ml a statistically significant pro-inflammatory response was observed during the 24 and 48 h exposure periods. This is in line with current literature regarding copper NP toxicity [17, 26, 27, 45–47], and confirmed that the CuO NPs used in this study are highly capable of inducing strong pro-inflammatory conditions and inducing substantial cell death. As we had shown relatively rapid dissolution of CuO NPs in CCM, although not a main focus of this study, we also addressed whether the biological responses described above can be attributed solely to Cu^2+^ toxicity, or whether copper presented in the form of NPs can exacerbate biological responses. This was tested by comparing the CuO NPs and CuCl_2_ at the same copper concentrations, and assessing the response of A549 cells in terms of cell death, viability and secretion of the pro-inflammatory cytokine IL-8. CuO NPs at the same copper concentrations as CuCl_2_ would consistently and significantly reduce A549 cell viability, increase cytotoxicity and increase pro-inflammatory mediator release to a greater extent than CuCl_2_. This effect has often been reported [24, 28, 48, 49] and is postulated as the Trojan horse mechanism [31], in which large quantities of copper are delivered into the cell in the form of particles, with subsequent rapid dissolution within acidic lysosomes [50], which, when present at high concentrations, Cu^2+^ ions may induce formation of reactive oxygen species, induce DNA strand breaks, and lipid peroxidation [51]. However, to distinguish discernible differences within the cells’ metabolome dependent upon whether the insult is via copper particles or Cu^2+^ ions is outside the scope of this study.

The dose- and time-dependent effects of CuO NPs on A549 cell death and inflammation were used to select concentrations and exposure times appropriate for the untargeted metabolomics profiling assays. As we desired to investigate sublethal concentrations, which would still elicit a measureable adverse response, we selected a final concentration of 10 μg/ml to be used in our metabolomics approach, with maximum exposure times of 24 h.

### Biological interpretation and validation of metabolomics – focus on oxidative stress markers

The metabolomics approach revealed a depletion of the reduced form of glutathione (GSH) and an increase in the oxidized form of glutathione (glutathione disulfide, GSSG) upon CuO NP treatment of A549 cells, this, accompanied with an increase in cysteine-glutathione disulfide, was indicative of antioxidant activity [52–54] and therefore oxidative stress. This was further corroborated by the elevated levels of citrulline, a product of nitric oxide formation [55–57]. Therefore, the first hypothesis to be generated upon the metabolomics approach was that CuO NPs induce oxidative stress. This is a process repeatedly reported concerning biological responses to CuO NPs [29, 30, 32, 45], and was validated within our study using a more conventional detection of glutathione oxidation and reduction states, and mRNA expression of antioxidants. A clear dose- and time-dependent increase in GSSG was found, indicating a significant oxidation of glutathione. In terms of the concentration used for the metabolomics study, 10 μg/ml, it was evident that a significant oxidation of glutathione only occurred after 6 h. This aligns relatively well with the data generated from assessing the cells’ whole metabolome; although the metabolomics data may provide a more sensitive assessment as, although significant differences in GSH were also observed here at 6 h, GSSG was found to significantly increase from 3 h onwards.

When assessing antioxidant gene expression, it was observed that transcription of both HO-1 and GPX1 genes occurred in response to CuO NPs. However, HO-1 gene expression occurred at lower particle concentrations and at earlier time points. Although activity of HO-1 is well reported in response to Cu NPs [46, 58, 59], GPX1 is less so, and in fact has been shown to decrease in response to Cu NPs [60]. Although dual assessment of HO-1 and GPX1 has not been performed elsewhere in CuO NP studies, the decreased sensitivity of GPX1 detection (or induction) can be corroborated in in vitro studies. Using CNTs exposures of mouse macrophages, an increase in HO-1 gene expression was found within 6 h in response to CNTs in the absence of GPX1 [61], a finding also observed by Chou et al. [62] in the treatment of human monocyte-derived macrophages with SWCNTs. This was further corroborated by Bussy et al. [63] who observed a rapid expression of HO-1, in the absence of GPX1, with exposure of mouse macrophages with CNTs, while prolonged exposures induced significant expression of both these markers [63]. Our data implies that HO-1 and glutathione oxidation are certainly more sensitive and earlier markers for CuO NP induced oxidative stress, compared to GPX1, with time kinetics of each coinciding, indicating that glutathione would be a suitable marker to use for metabolome screening.

### Biological interpretation and validation of metabolomics – focus on apoptosis markers

An additional goal in conducting this study, to determine potential markers of apoptosis, was more challenging to accomplish. We identified certain metabolites in response to CuO NPs which could be interpreted as being involved in cell death, including elevated levels of GPC, various amino acids, myo-inositol, and MTA; all of which have been associated with hypertonic stress and/or apoptosis [64–72]. The potential of CuO NPs to induce hypertonic stress is in itself an interesting point to consider. The relatively specific signaling cascades, which can occur in response to changes in osmolarity, may provide novel understanding of toxicity mechanisms induced by CuO NPs and NPs in general. For example, the activation and phosphorylation of the Rel family protein NFAT5 is induced by hypertonicity [66], as is NF-kB phosphorylation [73]. Activation of NFAT5 promotes pro-inflammatory responses such as TNF-α secretion in T cells [66], IL-1β and TNF-α secretion in epithelial cells [74]. This activity differs from other Rel proteins (NFATc), as activation of NFAT5 is thought to be independent from intracellular calcium-regulated effects [66]. As intracellular calcium regulation is a commonly reported mode of action in nanotoxicology [75–78], the possibility that CuO NPs employ mechanisms distinct from this, i.e. osmotic stress and NFAT5 activation, is an interesting observation. It is clear that these issues would need further study to distinguish if one or both mechanisms are relevant for the results found here. Furthermore, the regulation of NFAT5 derives not only from osmotic stress, but also from growth factors, cytokines, ROS, and ions, for example [79]. The association of CuO NPs and other metal NPs (with responses attributed to ion release) with osmotic effects has already been made in the assessment of microorganisms such as algae [80] and bacteria [81, 82], and in aquatic systems [83]. In mammalian nucleated cells, associations between metal NPs and osmotic stress-induced responses are far less reported. Gold NPs have been shown to induce transcription of genes associated with osmotic stress in HEp-2 cells [84].

All amino acids detected were shown to accumulate here upon treatment with CuO NPs over time, as was myo-inositol. This accumulation of amino acids [64, 65], and myo-inositol [66], have both previously been associated with hypertonic stress. Furthermore, as the events associated with osmotic stress, such as apoptotic volume decrease (AVD), have been reported to occur prior to many standard markers of apoptosis, such as caspase activity, cytochrome c release, or DNA laddering [85], it is plausible that this increase in amino acids residing intracellularly could be an early marker for the initiation of apoptosis. This is also supported by reports that amino acid accumulation (Gln especially) results in non-physiological conditions that play a decisive role in regulation of stress responses, such as encouraging heat shock proteins expression [86, 87]. We also observed an intracellular accumulation over time of GPC in response to CuO NP treatment of A549 cells, with the highest observed level (14-fold increase compared to untreated cells) after 24 h. GPC is a degradation product of phosphatidylcholine, and has also been long associated with protective functions during osmotic stress [67], has been reported in association with apoptosis [68–70], and as a biomarker for effects of NPs on biological systems [88]. MTA was also considered as a potential metabolome candidate marker for apoptosis. We found levels of MTA to significantly increase from 3 h onwards, and amongst several important cellular functions, including roles in gene expression and proliferation, MTA is considered to be involved in the regulation of, and even to promote, apoptosis [71, 72]. The rise in the aforementioned metabolites provides an indication of their involvement in apoptotic events induced by CuO NPs, and, moreover, the GPC found elevated here in response to CuO NPs has also been observed in response to other NPs elsewhere, such as gold nanorods [89]. Moreover, MTA levels were shown to decrease in keratinocytes in response to TiO_2_, while aptoptosis was not detected [90].

To validate whether these events may relate to apoptosis, and to confirm whether the CuO NPs tested here induce apoptosis, a more conventional apoptosis assay was performed as comparison, by measuring the activity of caspase-3 and −7. These are cysteine proteases known to play mediatory roles in both mitochondrial-mediated and death receptor-mediated apoptosis [91]. This assay was performed in conjunction with further untargeted metabolomics and determination caspase-3 and −7 activity in A549 cells in response to STS and CPT, to further clarify the involvement of our identified metabolites in apoptosis, and to gain some assurance of their strength as generic markers for apoptosis. Both STS and CPT are known to induce apoptosis [92, 93], and were shown to do so here.

The increase of specific metabolites we perceived to be indicative of CuO NP-induced apoptosis was often dissimilar when comparisons were made to STS and CPT induced responses. MTA was unaffected by STS and CPT. While GPC was elevated in response to CPT over time, the response to STS did not follow any time-dependent responses. With other metabolites also not following a strict conformity between treatments, such as glutathione, sphingosine, and phosphocreatine, these findings infer that the differential metabolite pattern of CuO NP treated cells was often dissimilar to STS and CPT. Furthermore, excluding amino acid levels (which were consistent across treatment methods), the previously determined metabolites GPC and MTA, although potential markers for CuO NP induced apoptosis, were considered to be inappropriate generic markers for apoptotic events. However, the strong time-dependent upregulation of MNA in response to all three treatments highlights this metabolite a potential generic screening option for apoptosis.

MNA is formed of nicotinamide and s-adenosylmethionine (SAMe), through the transfer of the methyl group of SAMe to nicotinamide by nicotinamide N-methyltransferase (NNMT) [94]. MNA detection has been used for disease diagnostic purposes, such as in determination of atherosclerosis [95]. Furthermore, although previously thought of as an inactive metabolite, MNA is now considered to be an active metabolite of nicotinamide, and its anti-inflammatory activities have led to its use in the treatment of inflammatory conditions such as thrombolysis [96] and rosacea [97]. In terms of NPs, MNA levels have been altered, in vivo, in response to iron oxide and manganese NPs [98–100]. Although MNA upregulation has not previously been reported in response to CuO NPs, there is significant inference of its effect in the present study, as MNA has previously been shown to induce DNA fragmentation in HL-60 cells [101]. All three treatments used here, CuO NPs, STS, and CPT, have been shown to induce significant increases in this metabolite, and all have also been shown to induce DNA strand breaks elsewhere [24, 28, 92, 93], it is, therefore, possible that MNA could be presented as a marker for DNA damage which leads to apoptosis. To confirm this proposed mode of action, absolute quantification of MNA in the cell lysates would be necessary and the determined concentration used for treatment of A549 cells, with subsequent measurements of DNA strand break; this, however, is beyond the scope of this study and a matter of ongoing research.

### Scope of untargeted metabolomics in nanosafety testing

For untargeted metabolomics methods to be of use in nanotoxicological testing, a number of points need to be considered: i) can untargeted metabolomics be considered a more sensitive method for determining NM-induced biological responses, in comparison to more conventional methods? ii) Is there extra information to be gained from performing untargeted metabolomics in comparison to conventional methods, and how can it be used?

I) It is important to assess the sensitivity of techniques such as those used here when establishing the usefulness and appropriateness of employing untargeted metabolomics in nanosafety assessment. When comparing the statistically significant regulation of A549 cells’ metabolome in response to CuO NPs to the responses observed, at the same particle concentration, using the more conventional methods a number of observations can be made. In terms of markers for oxidative stress, a significant alteration of GSH/GSSG ratios was observed at 6 h using the conventional method, and mRNA expression of antioxidants (HO-1) was increased also at 6 h. Markers for oxidative stress within the metabolome were at times decidedly different. The decline of GSH was only observed to be significant after 12 h, the same observation was made for citrulline. However, cysteine glutathione disulfide was elevated at 6 h, and the increase in oxidised glutathione (GSSG) was statistically significant after just 3 h. This highlights that the breadth of information gathered with metabolomics allows earlier and therefore more sensitive detection of oxidative stress induced by CuO NPs.

The same distinction in sensitivity can be made in terms of apoptosis; using conventional assessment of caspase-3 and −7 activity we observed a significant increase after 12 h exposure of 10 μg/ml. At this same concentration, markers perceived to be associated with apoptosis were altered time-dependently: GPC increased after 12 h, myo-inositol after 6 h, while MNA, MTA and Gln were all significantly increased after just 3 h. Again highlighting that the breadth of features detected allowed for a more sensitive assessment.

II) There is currently a growing opinion that the way in which toxicology is assessed for regulatory purposes is not fully sufficient [102]. This is based on limitations of animal models as comparisons to humans, but also of the need of in vivo studies to often use maximum tolerated doses, to remain time- and cost-effective [102]. Furthermore, with the exponential growth of nanotechnology and materials pertaining to it, new schemes and alternative testing strategies which operate within the 3Rs principle are essential, and in current times ethically prudent [103, 104]. For example, REACH are encouraging an integrated approach to toxicological testing, where in vivo, in vitro and in silico methods are used [102]. To be of use, it is proposed that the relevant data be incorporated into conceptual schemes, such as adverse outcome pathways (AOPs), which allow the linking of early molecular events to the eventual adverse outcome in an organism [105]. Part of these initiatives involve the determination of modes of action, which is where use of omics techniques, such as metabolomics, is considered paramount [102]. Metabolomics has developed rapidly as a new discipline to be used within these schemes and offers an opportunity for a screening approach, to discover new metabolites that are effected upon NP treatments.

The focus of this present study was to establish whether untargeted metabolomics could identify markers of key toxicity pathways associated with CuO NPs. Here, in this proof of principle study, we have demonstrated that metabolomics can detect specific markers of both oxidative stress and apoptosis, and that this method also allows the distinction between molecules involved in the apoptotic pathways of mechanistically different stimuli (CuO NPs, STS, and CPT), but also those molecules found to have homogeneity across different stimuli. At this stage we are confident in the techniques which have been used, and in our propositions for pathway markers. It is beyond the scope of one study to evaluate all of the pathways implied by this analysis of the cells’ whole metabolome. Including the potentially novel CuO NP toxicity mechanisms associated with changes in osmolarity, the assessment of agents contributing or causing DNA damage, determination of whether a response to copper particulates differs from copper ions, or whether copper particles of different shapes or sizes can affect the cells’ metabolome in different fashions. These questions remain important for ongoing research, as there are very few published metabolomics studies focusing on NPs.

To the best of our knowledge, no other study has determined the metabolic profile in response to CuO NPs, or NPs in general, in lung epithelial cells in vitro. Of the current published research there are few studies which have determined the metabolic profile induced by other NPs and/or in other systems, such as the copper NP exposure of rats by oral gavage [9]. Furthermore, with intravenous exposures of nano- and submicron-sized silica particles a metabolic analysis of liver toxicity demonstrated that both particle sizes induced a similar metabolite profile [106]. However, the main focus of metabolomics in nanosafety testing has been on TiO_2_. Including the assessment of earthworms [11], intratracheal or intragastric exposure of TiO_2_ to rats [107, 108], and various mammalian cell in vitro responses to TiO_2_ [109–111], but also the in vitro exposure of Caco-2 cells with gold NPs of different sizes, in which the different sized Au NPs were shown to induce similar metabolic profiles [112]. It is unclear whether Cu^2+^ would exert the same effect as we have observed for CuO NPs, or even whether other metal oxide NPs would do so. For example, in a study by Garcia et al. [110], TiO_2_ induced a different metabolite profile in fibroblasts than the one observed here. Those metabolites which were increased and implicated in toxic responses here (MNA, GPC, and amino acids) were found to decrease in response to treatments of TiO_2_ alone [110], which were of a similar size to the CuO NPs used here. While levels of glutathione where unchanged in response to TiO_2_ [110], CuO NPs decreased GSH here. Conversely, in HepG2 cells TiO_2_, again of a similar size, were shown to reduce glutathione levels [109]. However, again, our metabolites of note (MTA and GPC) were unchanged in TiO_2_ exposures (only exposures of TiO_2_ of a relatively larger size (~100 nm) induced GPC elevation); MNA was not reported [109].

## Conclusion

We assessed the toxicity mechanism of CuO NPs in A549 cells and have found that it involves strong pro-inflammatory conditions, oxidative stress, and programmed cell death, accompanied by a significant alteration of the cells’ metabolic activity. Toxicity of CuO NPs has been shown to be greater than that of corresponding concentrations of Cu^2+^ ions. To the best of our knowledge, no other study has performed a CuO nanotoxicological/metabolomics study to this technical level; specifically, we focused on thorough quality control and stringent validation of the metabolome data by carrying out dedicated conventional cellular and biochemical assays in order to provide a subsequent critical testing of the hypotheses generated by HPLC-MS, which adds great strength to the suppositions of this research. The untargeted metabolomics approach was shown to be a particularly sensitive method, and able to detect subtle metabolic changes in A549 cells upon exposure of CuO NPs. Although not absolute, relative quantification was possible, and it has been shown here to provide, in one experiment, an answer that would normally need multiple dedicated cell assays for specific endpoints; highlighting untargeted metabolomics as a suitable tool for the high-throughput screening of NPs.

Ultimately, only the normalization of metabolic expression patterns to decreasing cell numbers upon treatment with toxic substances allows an unbiased view. Thus it is mandatory to apply suitable normalization parameters. For untargeted metabolomics to be used as a suitable high-throughput screening tool, however, it is important to obtain strong, reproducible markers, in this case for pathways which are well reported in CuO NP induced toxicity, oxidative stress and apoptosis. As expected, the well-recognized marker for oxidative stress, ratios of reduced and oxidized glutathione, was established in both metabolic and conventional detection assays, while assured markers for apoptosis were more difficult to identify. Ultimately, MNA is tentatively suggested as a strong metabolic marker for apoptosis, as it not only was found in response to CuO NPs but also other compounds which use mechanistically distinct pathways of apoptosis induction. This hypothesis will need further testing and validation with a broader range of NPs. However, we have demonstrated here that the use of metabolomics in this fashion can provide molecular mapping of toxicity pathways, which can contribute to the development of AOPs, but also can be used as a tool for hypotheses generation and toxicity screening.

## Additional file

Additional file 1: Table S1. Tuning parameters QExactive Plus. **Table S2.** Cell numbers [x10^6^] for Control (C) as well as CuO NP Treated (T). **Table S3.** Cell numbers [x10^6^] of Control (C), STS treated (STS) and Camptothecin treated (CPT) for the different time points. **Table S4.** Relative standard deviation (RSD) of internal standards for metabolomics profiling – values for Ethylparabene and Nitrotyrosine in CuO NP profiling. **Table S5.** Feature number after the different steps of data processing. **Figure S1.** Overlaps at feature level for RP and HILIC separation for (a) positive ESI-mode and (b) negative ESI-mode. **Figure S2.** Cell viability, cytotoxicity, and IL-8 secretion in response to CuO NPs and Cl2Cu•2H2O. **Table S6.** Differentially regulated metabolites upon CuO NP treatment. **Table S7.** Number of differentially expressed features found for each separation method (reversed phase as well as hydrophilic interaction chromatography in positive and negative modes). **Figure S3.** HO-1 gene expression in response to CuO NPs. **Figure S4.** GPX1 gene expression in response to CuO NPs. **Figure S5.** Bar plot of peak areas for Methylnicotinamide at the time points (0 h, 6 h, 12 h and 24 h) of both metabolomics studies CuO NP (control CuO study and CuO treatment) and apoptosis study (control apoptosis study, STS treatment and CPT treatment). (DOCX 193 kb)

## Abbreviations

ACN: Acetonitrile
AOP: Adverse outcome pathway
AVD: Apoptotic volume decrease
CCM: Cell culture medium
CPT: Camptothecin
CTB: Celltiter-Blue
CuO NPs: Copper oxide nanoparticles
DLS: Dynamic light scattering
FCS: Foetal calf serum
GPC: Glycerophosphocholine
GPX1: Glutathione peroxidase 1
GSH: Reduced glutathione
GSSG: Oxidized glutathione
HILIC: Hydrophilic interaction liquid chromatography
HMDB: Human metabolite database
HO-1: Heme oxygenase 1
HPLC-MS: High-performance liquid chromatography-mass spectrometry
ICP-AES: Inductively coupled plasma atomic emission spectroscopy
IL-8: Interleukin-8
LDH: Lactate dehydrogenase
LIMMA: Linear models for microarray data
MNA: Methylnicotinamide
MS^2^: Tandem mass spectrometry
MTA: Methylthioadenosine
NPs: Nanoparticles
PBS: Phosphate buffered saline
PCA: Principal component analysis
RP-HPLC: Reversed-phase high-performance liquid chromatography
RSD: Relative standard deviation
STS: Staurosporine
TEM: Transmission electron microscopy
TNF-α: Tumour necrosis factor-alpha
