# Supplementary material for: Copper oxide nanoparticle toxicity profiling using untargeted metabolomics
Source: Part Fibre Toxicol. 2016 Sep 8;13:49. doi: 10.1186/s12989-016-0160-6 (PMC5017021; doi:10.1186/s12989-016-0160-6)
Supplement: Additional file 1: Table S1. — Tuning parameters QExactive Plus. Table S2. Cell numbers [x106] for Control (C) as well as CuO NP Treated (T). Table S3. Cell numbers [x106] of Control (C), STS treated (STS) and Camptothecin treated (CPT) for the different time points. Table S4. Relative standard deviation (RSD) of internal standards for metabolomics profiling – values for Ethylparabene and Nitrotyrosine in CuO NP profiling. Table S5. Feature number after the different steps of data processing. Figure S1. Overlaps at feature level for RP and HILIC separation for (a) positive ESI-mode and (b) negative ESI-mode. Figure S2. Cell viability, cytotoxicity, and IL-8 secretion in response to CuO NPs and Cl2Cu•2H2O. Table S6. Differentially regulated metabolites upon CuO NP treatment. Table S7. Number of differentially expressed features found for each separation method (reversed phase as well as hydrophilic interaction chromatography in positive and negative modes). Figure S3. HO-1 gene expression in response to CuO NPs. Figure S4. GPX1 gene expression in response to CuO NPs. Figure S5. Bar plot of peak areas for Methylnicotinamide at the time points (0 h, 6 h, 12 h and 24 h) of both metabolomics studies CuO NP (control CuO study and CuO treatment) and apoptosis study (control apoptosis study, STS treatment and CPT treatment). (DOCX 193 kb) [file 12989_2016_160_MOESM1_ESM.docx]

Additional file 1: contains seven supplementary tables and five supplementary figures.

Journal: Particle and Fibre Toxicology

Copper oxide nanoparticle toxicity profiling using untargeted metabolomics

Matthew S.P. Boyles^1*^, Christina Ranninger^2*^, Roland Reischl^2^, Marc Rurik^3,4^, Richard Tessardri^5^, Oliver Kohlbacher^3,4,6,7,8^, Albert Duschl^1†^, Christian G. Huber^2†^.

^1^ Department of Molecular Biology, Division of Allergy and Immunology, University of Salzburg, Hellbrunner Strasse 34, 5020 Salzburg, Austria

^2^ Department of Molecular Biology, Division of Chemistry and Bioanalytics, University of Salzburg, Hellbrunner Strasse 34, 5020 Salzburg, Austria

^3^ Center for Bioinformatics, University of Tübingen, Tübingen, Germany

^4^ Dept. of Computer Science, University of Tübingen, Germany

^5^ Faculty of Geo- and Atmospheric Science, University of Innsbruck, Innrain 52, 6020 Innsbruck, Austria

^6^ Quantitative Biology Center, University of Tübingen, Germany

^7^ Faculty of Medicine, University of Tübingen, Germany

^8^ Biomolecular Interactions, Max Planck Institute for Developmental Biology, Tübingen, Germany

* Denotes co-first authors. Matthew Boyles and Christina Ranninger have contributed equally to this work.

† Denotes co-corresponding authors:

Albert Duschl Tel: +43 662 8044 5730, Fax: +43 662 8044 5751, Email: [albert.duschl@sbg.ac.at](mailto:albert.duschl@sbg.ac.at)

Christian G. Huber, Tel: +43 662 8044 5738, Fax: +43 662 8044 5751, Email: [c.huber@sbg.ac.at](mailto:c.huber@sbg.ac.at)

Table S1. Tuning parameters QExactive Plus

| MS-Parameter | Q Exactive Plus | | | |
| --- | --- | --- | --- | --- |
|  | **RP_pos** | **RP_neg** | **HILIC_pos** | **HILIC_neg** |
| Sheath gas | 25 | 35 | 20 | 20 |
| Aux gas | 5 | 5 | 10 | 10 |
| Spray voltage[kV] | 3 | 3 | 3.5 | 3.5 |
| Capillary temperature [°C] | 320 | 350 | 200 | 250 |
| S-lens | 50 | 50 | 50 | 50 |

Table S2. Cell numbers [x10^6^] for Control (C) as well as CuO NP Treated (T)

| Time [h] | C_1 | C_2 | C_3 | Mean (C) | T_1 | T_2 | T_3 | Mean (T) |
| --- | --- | --- | --- | --- | --- | --- | --- | --- |
| 0 | 3.15 | 3.07 | 3.13 | 3.12 | 2.84 | 3.01 | 2.775 | 2.88 |
| 1 | 2.79 | 2.9 | 3.92 | 3.20 | 3.25 | 2.75 | 4.05 | 3.35 |
| 3 | 3.79 | 2.8 | 3.04 | 3.21 | 2.64 | 2.8 | 3.07 | 2.84 |
| 6 | 3.83 | 2.99 | 3.04 | 3.29 | 3.51 | 2.71 | 2.6 | 2.94 |
| 12 | 3.31 | 3.86 | 3.56 | 3.58 | 2.48 | 2.68 | 2.44 | 2.53 |
| 24 | 5.2 | 5.39 | 4.88 | 5.16 | 1.69 | 2.65 | 1.61 | 1.98 |

Table S3. Cell numbers [x10^6^] of Control (C), STS treated (STS) and Camptothecin treated (CPT) for the different time points

| Time [h] | C_1 | C_2 | C_3 | Mean  C | STS_1 | STS_2 | STS_3 | Mean  STS | CPT_1 | CPT_2 | CPT_3 | Mean  CPT |
| --- | --- | --- | --- | --- | --- | --- | --- | --- | --- | --- | --- | --- |
| 0 | 2.48 | 3.16 | 3.27 | 2.97 | 2.51 | 4.02 | 2.84 | 3.12 | 2.67 | 3.35 | 2.55 | 2.86 |
| 3 | 2.89 | 2.97 | 2.93 | 2.93 | 2.59 | 2.81 | 2.61 | 2.67 |  |  |  |  |
| 6 | 4.34 | 3.26 | 3.28 | 3.63 | 2.65 | 2.64 | 2.71 | 2.67 | 3.21 | 2.72 | 3.6 | 3.18 |
| 9 | 4.33 | 3.18 | 3.73 | 3.75 |  |  |  |  | 3.33 | 3.2 | 3.5 | 3.34 |
| 12 | 5.39 | 3.8 | 3.77 | 4.32 | 2.45 | 2.32 | 2.07 | 2.28 | 2.67 | 2.66 | 3.14 | 2.82 |
| 24 | 5.01 | 4.85 | 5.06 | 4.97 | 2.21 | 2.05 | 1.6 | 1.95 | 1.78 | 1.77 | 1.78 | 1.78 |

Table S4. Relative standard deviation (RSD) of internal standards for metabolomics profiling – values for Ethylparabene and Nitrotyrosine in CuO NP profiling

|  | Intracellular  RP  Positive [%] | Intracellular  RP  Negative [%] | Intracellular  HILIC  Positive [%] | Intracellular  HILIC  Negative [%] | Apoptosis  RP  Positive [%] | Apoptosis  HILIC  Negative [%] |
| --- | --- | --- | --- | --- | --- | --- |
| Ethylparabene | n.d | 12.99 | n.d | 8.80 | n.d. | 14.00 |
| Nitrotyrosine | 10.40 | 10.87 | 11.45 | 8.22 | 9.69 | 16.57 |

Table S5. Feature number after the different steps of data processing

|  | Intracellular  RP  Positive | Intracellular  RP  negative | Intracellular  HILIC  Positive | Intracellular  HILIC  Negative |
| --- | --- | --- | --- | --- |
| No. after feature linking (OpenMS) | 2011 | 6220 | 1397 | 2293 |
| No. after Blank filtering (KNIME) | 137 | 291 | 245 | 597 |
| No. after QC filtering (KNIME) | 113 | 144 | 206 | 495 |

Figure S1. Overlaps at feature level for RP and HILIC separation for (a) positive ESI-mode and (b) negative ESI-mode.

 **Figure S2. Cell viability, cytotoxicity, and IL-8 secretion in response to CuO NPs and Cl_2_Cu·2H_2_O.** A549 cells treated with CuO NPs (dark bars) or Cl_2_Cu·2H_2_O (light bars) at corresponding copper concentrations of 63, 125, 250 and 500 µM for 4, 24 and 48 h and assessed for cell viability (top row), LDH release (middle row), and IL-8 secretion (bottom row). Each data point represents the mean ± SEM of three independent biological replicates. Statistical significant differences were determined by independent sample T-Test, and identified with * = p<0.05, ** = p<0.01, or *** = p<0.005 when comparing CuO NPs and Cl_2_Cu·2H_2_O.

Table S6. Differentially regulated metabolites upon CuO NP treatment.

^1)^Compound ID was established by accurate mass, fragment spectra and retention time of a reference substance (Level 1); ^2)^Compound ID was established by accurate mass and retention time of a reference substance (Level 2); ^a)^p-value <0.05 at least for one of H03 or H14; values are log2 FC ratios relative to controls (according to standards proposed in ([Sumner et al. 2007](#_ENREF_1))).

| m/z | RT [s] | ID | p_0h | p_1h | p_3h | p_6h | p_12h | p_24h | FC_0h | FC_1h | FC_3h | FC_6h | FC_12h | FC_24h |
| --- | --- | --- | --- | --- | --- | --- | --- | --- | --- | --- | --- | --- | --- | --- |
| 116.0702 | 684 | HMDB00162 (L-Proline; M+H); HILIC^1^ | 0.57464 | 0.77052 | 0.00316 | 0.00092 | 0.00134 | 0.00002 | 1.10 | 0.95 | 1.45 | 2.25 | 2.27 | 2.02 |
| 120.0651 | 49 | HMDB00167 (L-Threonine; M+H); RP^1^ | 0.41571 | 0.84553 | 0.08904 | 0.00250 | 0.01304 | 0.00017 | 1.12 | 1.13 | 1.35 | 2.62 | 3.72 | 4.01 |
| 130.0510 | 724 | HMDB00725 (Hydroxyproline; M-H); HILIC^1^ | 0.83522 | 0.97430 | 0.02000 | 0.00552 | 0.00065 | 0.00118 | 1.06 | 1.02 | 1.53 | 2.47 | 3.24 | 3.32 |
| 130.0510 | 306 | HMDB00766 (N-Acetyl-L-alanine; M-H); HILIC^1^ | 0.56450 | 0.89940 | 0.48310 | 0.03719 | 0.00218 | 0.00041 | 1.08 | 0.94 | 1.05 | 0.73 | 0.61 | 0.52 |
| 132.0763 | 744 | HMDB00064 (Creatine; M+H); RP^1^ | 0.55202 | 0.58260 | 0.70464 | 0.06229 | 0.01308 | 0.00062 | 1.16 | 1.05 | 1.06 | 0.74 | 0.58 | 0.36 |
| 132.1015 | 112 | HMDB00687 (L-Leucine; M+H); RP^1^ | 0.17550 | 0.94062 | 0.01341 | 0.00114 | 0.00002 | 0.00023 | 1.21 | 1.03 | 1.54 | 2.87 | 3.41 | 4.32 |
| 132.1015 | 104 | HMDB00172 (L-Isoleucine; M+H); RP^1^ | 0.40739 | 0.55187 | 0.01863 | 0.00140 | 0.00000 | 0.00024 | 1.16 | 1.09 | 1.45 | 2.75 | 3.53 | 3.77 |
| 133.0603 | 764 | HMDB00168 (L-Asparagine; M+H); HILIC^1^ | 0.71420 | 0.83046 | 0.03507 | 0.00005 | 0.00031 | 0.00007 | 1.08 | 1.06 | 1.59 | 3.04 | 4.01 | 4.07 |
| 137.0705 | 51 | HMDB03152 (N-Methylnicotinamide; M+H); RP^1^ | 0.98516 | 0.55187 | 0.01908 | 0.00292 | 0.00093 | 0.00015 | 0.99 | 1.50 | 2.86 | 3.15 | 9.07 | 28.30 |
| 147.0759 | 759 | HMDB00641 (L-Glutamine; M+H); HILIC^1^ | 0.64145 | 0.75690 | 0.00175 | 0.00007 | 0.00274 | 0.00003 | 1.10 | 1.04 | 1.59 | 2.92 | 3.56 | 3.20 |
| 146.0460 | 49 | HMDB00148 (L-Glutamic acid; M-H); RP^1^ | 0.70312 | 0.85441 | 0.75541 | 0.27670 | 0.01172 | 0.00151 | 1.10 | 1.07 | 1.04 | 1.26 | 1.49 | 2.15 |
| 146.1171 | 54 | HMDB06831 (3-Dehydroxycarnitine; M+H); RP^2^ | 0.29597 | 0.84553 | 0.08691 | 0.54773 | 0.06241 | 0.01335 | 1.13 | 0.98 | 1.13 | 1.08 | 1.13 | 0.80 |

| m/z | RT [s] | ID | p_0h | p_1h | p_3h | p_6h | p_12h | p_24h | FC_0h | FC_1h | FC_3h | FC_6h | FC_12h | FC_24h |
| --- | --- | --- | --- | --- | --- | --- | --- | --- | --- | --- | --- | --- | --- | --- |
| 150.0578 | 609 | HMDB00696 (L-Methionine; M+H); HILIC^1^ | 0.46462 | 0.84966 | 0.02802 | 0.00085 | 0.00046 | 0.00006 | 1.36 | 0.94 | 1.50 | 3.41 | 4.59 | 7.09 |
| 151.0262 | 161 | HMDB00292 (Xanthine; M-H); HILIC^1^ | 0.49382 | 0.74828 | 0.98728 | 0.72354 | 0.11954 | 0.00007 | 1.25 | 0.85 | 1.00 | 0.90 | 1.20 | 4.42 |
| 166.0857 | 244 | HMDB00159 (L-Phenylalanine; M+H); RP^1^ | 0.16341 | 0.86567 | 0.00336 | 0.00140 | 0.00002 | 0.00011 | 1.23 | 1.07 | 1.50 | 2.71 | 3.81 | 4.47 |
| 174.0886 | 50 | HMDB00904 (Citrulline; M-H); RP^1^ | 0.87458 | 0.99790 | 0.48571 | 0.23701 | 0.01027 | 0.00564 | 0.76 | 1.00 | 1.33 | 2.27 | 3.70 | 3.37 |
| 176.0388 | 205 | HMDB01015 (N-Formyl-L-methionine; M-H); RP^1a^ | 0.86777 | 0.65966 | 0.14709 | 0.04129 | 0.00305 | 0.00021 | 1.04 | 0.89 | 1.41 | 1.38 | 2.00 | 4.64 |
| 181.0602 | 268 | HMDB03269 (Nicotinuric acid; M+H); RP^1a^ | 0.49308 | 0.94062 | 0.18054 | 0.26182 | 0.02341 | 0.00041 | 1.12 | 1.04 | 1.21 | 1.15 | 1.52 | 2.64 |
| 182.0805 | 632 | HMDB00158 (L-Tyrosine; M+H); HILIC^1^ | 0.89185 | 0.74732 | 0.04494 | 0.00286 | 0.00291 | 0.00054 | 1.20 | 0.90 | 1.54 | 2.56 | 3.88 | 4.62 |
| 188.1751 | 1052 | HMDB01276 (N1-Acetylspermidine; M+H); HMDB:HMDB02189 (N8-Acetylspermidine; M+H); HILIC² | 0.49115 | 0.84966 | 0.16891 | 0.00255 | 0.02774 | 0.00844 | 1.45 | 1.07 | 2.11 | 2.33 | 3.40 | 9.76 |
| 192.0668 | 163 | HMDB00821 (Phenylacetylglycine; M-H); HILIC² | 0.49382 | 0.99249 | 0.02627 | 0.01524 | 0.00070 | 0.00041 | 1.19 | 1.01 | 2.07 | 2.10 | 4.21 | 15.29 |
| 205.0965 | 282 | HMDB00929 (L-Tryptophan; M+H); RP^1^ | 0.41722 | 0.76157 | 0.15759 | 0.00279 | 0.00085 | 0.00005 | 1.10 | 1.13 | 1.43 | 2.66 | 3.37 | 4.53 |
| 212.0423 | 49 | HMDB01511 (Phosphocreatine; M+H); RP^1^ | 0.93806 | 0.94062 | 0.81707 | 0.35138 | 0.00533 | 0.00017 | 1.02 | 0.99 | 1.03 | 0.86 | 0.62 | 0.39 |
| 220.1172 | 263 | HMDB00210 (Pantothenic acid; M+H); RP^1^ | 0.97155 | 0.76157 | 0.30176 | 0.78956 | 0.03556 | 0.00156 | 0.99 | 1.16 | 1.08 | 0.96 | 1.24 | 1.88 |
| 258.1091 | 875 | HMDB00086 (Glycerophosphocholine; M+H), HILIC^1^ | 0.54073 | 0.72793 | 0.66500 | 0.56603 | 0.02246 | 0.00008 | 1.29 | 1.47 | 1.14 | 1.18 | 2.74 | 13.99 |

| m/z | RT [s] | ID | p_0h | p_1h | p_3h | p_6h | p_12h | p_24h | FC_0h | FC_1h | FC_3h | FC_6h | FC_12h | FC_24h |
| --- | --- | --- | --- | --- | --- | --- | --- | --- | --- | --- | --- | --- | --- | --- |
| 298.0956 | 125 | HMDB01173 (5'-Methylthioadenosine; M+H); HILIC^1^ | 0.35560 | 0.72793 | 0.02802 | 0.00564 | 0.00291 | 0.00006 | 1.15 | 0.83 | 1.56 | 1.96 | 2.16 | 5.25 |
| 300.2886 | 523 | HMDB00252 (Sphingosine; M+H); RP^1^ | 0.91981 | 0.94062 | 0.11299 | 0.01530 | 0.00920 | 0.00201 | 1.03 | 1.08 | 0.81 | 0.55 | 0.57 | 0.49 |
| 306.0769 | 836 | HMDB00125 (Glutathione; M-H);ref HILIC^1^ | 0.98423 | 0.74828 | 0.19888 | 0.05420 | 0.00439 | 0.07643 | 1.00 | 0.91 | 0.86 | 0.63 | 0.49 | 0.73 |
| 306.0771 | 74 | HMDB00125 (Glutathione; M-H);ref RP^1^ | 0.84788 | 0.99790 | 0.41418 | 0.34320 | 0.00938 | 0.02177 | 0.78 | 1.01 | 0.68 | 0.49 | 0.12 | 0.16 |
| 333.0596 | 832 | HMDB11649 (1-(sn-Glycero-3-phospho)-1D-myo-inositol; M-H); HILIC² | 0.69718 | 0.42085 | 0.05808 | 0.00840 | 0.00058 | 0.00002 | 1.09 | 1.14 | 1.35 | 1.55 | 2.52 | 4.39 |
| 425.0815 | 53 | HMDB00656 (Cysteineglutathione disulfide; M-H); RP² | 0.70312 | 0.53015 | 0.48571 | 0.05744 | 0.00190 | 0.00151 | 1.65 | 0.78 | 1.15 | 3.77 | 6.87 | 6.93 |
| 427.0938 | 53 | HMDB00656 (Cysteineglutathione disulfide; M+H); RP² | 0.16341 | 0.55187 | 0.31834 | 0.00382 | 0.00145 | 0.00008 | 1.64 | 0.64 | 1.16 | 3.90 | 5.46 | 9.05 |
| 538.5178 | 61 | HMDB00790 (N-Palmitoylsphingosine; M+H); HMDB04949 (Ceramide (d18:1/16:0); M+H); HILIC² | 0.35560 | 0.72793 | 0.88002 | 0.04775 | 0.00291 | 0.00005 | 1.14 | 0.93 | 1.02 | 1.60 | 2.25 | 5.09 |
| 611.1461 | 1086 | HMDB03337 (Oxidized glutathione; M-H); HILIC^1^ | 0.66554 | 0.82074 | 0.01391 | 0.00552 | 0.00070 | 0.00287 | 1.58 | 1.38 | 2.97 | 5.78 | 7.95 | 11.84 |
| 613.1576 | 122 | HMDB03337 (Oxidized glutathione; M+H); RP^1^ | 0.25733 | 0.69410 | 0.81707 | 0.50891 | 0.82709 | 0.01113 | 1.28 | 1.32 | 1.04 | 0.87 | 1.03 | 1.65 |

Table S7. Number of differentially expressed features found for each separation method (reversed phase as well as hydrophilic interaction chromatography in positive and negative modes)

|  | Number of significantly regulated metabolites | | | |
| --- | --- | --- | --- | --- |
| **Time [h]** | **RP-pos** | **RP-neg** | **HILIC-pos** | **HILIC-neg** |
| 3h | 13 | 6 | 3 | 40 |
| 6h | 29 | 0 | 15 | 62 |
| 12h | 53 | 62 | 80 | 176 |
| 24h | 86 | 105 | 158 | 370 |
|  |  |  |  |  |

 **Figure S3. HO-1 gene expression in response to CuO NPs.** A549 cells treated with CuO NPs at 0 - 40 µg/ml. HO-1 mRNA expression was determined by qRT-PCR after 1, 3, 6, 12 and 24 h. Data is expressed in relation to the RPLP0 housekeeping gene and presented as fold increase compared to medium only treated cells. Each data point represents the mean ± SEM of three independent biological replicates. Statistical significance (determined by ANOVA with Tukey posthoc) is shown by * = p<0.05, ** = p<0.01, *** = p<0.005, when compared to medium only treated cells.

Figure S4. GPX1 gene expression in response to CuO NPs. A549 cells treated with CuO NPs at 0 - 40 µg/ml. GPX1 mRNA expression was determined by qRT-PCR after 1, 3, 6, 12 and 24 h. Data is expressed in relation to the RPLP0 housekeeping gene and presented as fold increase compared to medium only treated cells. Each data point represents the mean ± SEM of three independent biological replicates. Statistical significance (determined by ANOVA with Tukey posthoc) is shown by * = p<0.05, *** = p<0.005, when compared to medium only treated cells.


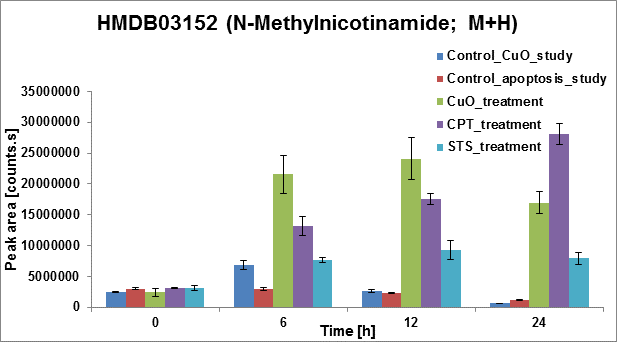


Figure S5. Bar plot of peak areas for Methylnicotinamide at the time points (0h, 6h, 12h and 24h) of both metabolomics studies CuO NP (control CuO study and CuO treatment) and apoptosis study (control apoptosis study, STS treatment and CPT treatment).

**References:**

Sumner L, Amberg A, Barrett D, et al. (2007) Proposed minimum reporting standards for chemical analysis. Metabolomics 3(3):211-221 doi:10.1007/s11306-007-0082-2
